# The impact of the health microinsurance M-FUND on the utilization of health services among migrant workers and their dependents in Thailand: A case-control study

**DOI:** 10.1016/j.jmh.2024.100236

**Published:** 2024-05-13

**Authors:** Andrea König, Jarntrah Sappayabanphot, Li Liang, Steffen Fleßa, Volker Winkler

**Affiliations:** a Epidemiology of Transition, Heidelberg Institute of Global Health, Heidelberg University Hospital, Im Neuenheimer Feld 130.3, Heidelberg 69120, Germany; b Faculty of Science, Asia Pacific International University, Saraburi, Thailand; c Faculty of Public Health, Thammasat University, Pathum Thani, Thailand; d Faculty of Law and Economics, University of Greifswald, Germany

**Keywords:** Migrant, Access to health care, Health microinsurance, Health coverage, Thailand

## Abstract

**Background:**

Even though Thailand has launched multiple migrant health policies, migrants still face various barriers in accessing health care. To some extent, these barriers may be reduced by health microinsurance. The M-FUND is a low-cost, not-for-profit health protection scheme for migrant workers in Thailand, created by Dreamlopments Social Enterprise and Foundation to support sustainable access to quality healthcare services for migrants. We aimed to investigate the impact of the M-FUND on utilization of health services.

**Methods:**

Over a period of three months, we conducted quantitative face-to-face interviews with 408 M-FUND members, and 452 age, sex and location matched non-member controls in Tak Province, Thailand. We compared utilization of health services between members and non-members using logistic regression controlling for a number of socio-demographic variables and variables related to migration such as years in Thailand, legal status, etc.

**Findings:**

M-FUND members were more likely to have utilized outpatient services (adjusted odds ratio: 1·74, 95 % confidence interval: 1·24–2·44), inpatient services (2·96, 1.11–7·92), and emergency care (1·89, 0·78–4·56), although the latter was not statistically significant. Members were more likely not to have purchased medicines over-the-counter (1·67, 1·22–2·27) than non-members. Members were also more likely to have utilized any additional specific service during a consultation (2·31, 1·49–3·58) including any type of imaging method (2·62, 1·29–5·29) and blood tests (1·64, 0·99–2·71). There were some differences between men and women, but most were not statistically significant.

**Interpretation:**

The M-FUND health microinsurance was positively associated with the utilization of all major types of health services among migrant workers and their dependents in Thailand. For migrants, particularly vulnerable migrant workers and their dependents, the M-FUND appears to be a good approach to reducing barriers to health care. This study of the impact of health microinsurance for migrants provides important information for policy and program planners in the field of migrant health. However, more research is needed on health microinsurance schemes for migrants in different settings and for other underserved communities as well as the sex-specific impact on health service utilization.

## 1 Introduction

Despite the fact that most migration takes place within low- and middle-income countries (LMICs), the focus continues to be on migration from LMICs to high-income countries (Abubakar et al., 2018). Many LMICs face multiple challenges in achieving universal health coverage (UHC) and policies that include (undocumented) migrants are rare, and where they exist, implementation is challenging (Abubakar and Zumla, 2018; Kodali, 2023). Health microinsurance has been proposed as a way to improve health coverage for migrants in settings with limited resources and overcome the challenges faced by government insurance schemes. In particular, access to certain groups of migrants, such as irregular, undocumented migrants, is difficult because they are not visible to governments. Health microinsurance providers may have better access to these populations through their community ties which build trust, help identify the uninsured and promote enrollment (Kimball et al., 2013). It was suggested that health microinsurance providers work with governments to overcome difficulties in accessing resources and achieving scale in order to help reach UHC (Kimball et al., 2013). However, there is limited experience with programs targeting migrants, and little research on their impact on UHC (Déglise, 2016; Wassink, 2018; Suphanchaimat et al., 2019; Wassink, 2016; Tschirhart et al., 2021).

Microinsurance is a risk pooling tool for low-income households (Churchill and Matul, 2012). It is used in various areas of life, including health, and can be operated by insurance companies, microfinance institutions, governments, or be community-based (Dror and Piesse, 2014). The term micro in microinsurance can refer to three things; (1) the financial situation of the clientele, i.e., a low-income population in developing countries; (2) the characteristics of the product, i.e., limited benefits for low premiums; and (3) the process by which the scheme is set up and managed (Dror and Piesse, 2014). Studies in the past have shown that health microinsurance can increase the probability of health care utilization among the poor in LMICs, such as Bangladesh, the Philippines or Senegal, in some cases with exemption from the poorest (Hamid et al., 2011; Jütting, 2004; Dror et al., 2005; Eze et al., 2023). Moreover, it can contribute to financial protection of insurance members (Eze et al., 2023; Habib et al., 2016), but it is faces a number of structural and managerial challenges (Heudtlass et al., 2016).

Due to the economic and demographic disparity with its neighboring countries Lao, Cambodia and Myanmar, Thailand attracts a large number of migrant workers, with approximately 1 to 2.5 million thought to be undocumented among the 4 to 5 million migrant workers estimated to be living in Thailand (International Organization for Migration, 2024a). Decades of conflict and/or lower economic development have increased the influx of labor migrants from Myanmar (Guinto et al., 2015; Suphanchaimat et al., 2017). The difficult conditions of migrant workers have been summarized in a scoping review of migrant health coverage in Thailand (König et al., 2022). While migrants are often healthier than the host population, they are at a higher risk of occupational injury and physical disability. They often face difficult living and working conditions and engage in unhealthy behaviours that increase their risk of mental health problems and non-communicable diseases (World Health Organization South-East Asia Region, 2018). In addition, precarious living conditions have been reported as exposing migrants to infectious diseases, such as malaria, tuberculosis and HIV/AIDS as well as to sexual exploitation and related violence (World Health Organization South-East Asia Region, 2018; UN Joint Programme on HIV/AIDS, 2014; Wangroongsarb et al., 2011; Tschirhart et al., 2017). These risks are even higher for undocumented migrants, and also victims of human trafficking, which is still common in Thailand (Suphanchaimat et al., 2017; International Organization for Migration, 2024b). Migrant women, about half of Thailand's migrant workers, also face challenges in accessing sexual and reproductive health services, which is associated with increased maternal mortality (Harkins, 2019).

The Thai government has issued several migrant health policies, and developed a migrant health insurance scheme, namely the Compulsory Migrant Health Insurance (CMHI), managed by the Ministry of Public Health. Moreover, the Social Security Scheme is accessible to some registered migrants (Guinto et al., 2015). Although these schemes are in principle available for registered migrants, there are still barriers to accessing health care and high out-of-pocket expenses. Many migrants never enroll or drop out of health insurance for a variety of reasons, including the limitation of coverage to a single hospital, which does not reflect the migrants’ unstable living conditions (Guinto et al., 2015), the requirement to pay two years’ fees in advance (for the CMHI), and the frequent changes in migration policies (Suphanchaimat et al., 2017). Undocumented migrants do not enroll due to lack of legal documents (Guinto et al., 2015), financial resources (Guinto et al., 2015; Suphanchaimat et al., 2017), and information (Prakunwisit and Areesantichai, 2015).

In 2017, the health protection scheme “Migrant-FUND” (hereafter M-FUND) was launched by the social enterprise and sister foundation “Dreamlopments” in cooperation with the Thai Ministry of Public Health (MOPH) to bridge the health insurance gap for migrants. M-FUND is a low-cost, not-for-profit health protection scheme that enrolls healthy migrants for a monthly fee of THB 100 (≈ US$ 2,85) (Dreamlopments, 2024) and covers overall broad healthcare services in partner hospitals, both for outpatient and inpatient services, up to the plan ceilings. It also offers tailored packages for pregnant women (THB 300/month), senior people above 50 years old (THB 200/month), and the chronically ill (THB 300/month). From launch in the city of Mae Sot (a major entry point for migrants from Myanmar) in Tak province, the protection scheme has expanded in Tak and other border provinces. As of June 2023, the M-FUND had enrolled approximately 59.000 migrants (Dreamlopments, 2019). An initial qualitative study suggests that this health protection scheme may have a positive impact on members’ health care utilization (Pudpong et al., 2019). A study focusing on maternal health suggests that the M-FUND contributes to including migrant women in universal health coverage (Tschirhart et al., 2021).

The objective of our research was to examine the impact of the M-FUND health protection scheme on health service utilization of migrant men and women in Thailand. Specifically, our research questions were: Did M-FUND members (a) utilize more outpatient services, inpatient services, and emergency care and buy fewer over-the-counter medicines? And (b) were they more likely to use additional services during a physician visit compared to non-members?

## 2 Methods

### 2.1 Study population and data

We conducted a case-control study between May and October 2021 in Tak Province, Thailand, in the M-FUND access areas of Mae Sot, Mae Ramat, and Phop Phra districts. All areas are located on the border between Thailand and Myanmar and are home to migrant workers and their families who have fled/moved from their country to escape insecurity or pursue economic opportunities (Sietstra, 2012). Data were collected through face-to-face interviews lasting approximately 60 min and were conducted using the Survey Solutions tablet-based software (The World Bank, 2018), mostly in participants’ homes. Interviewers had experience with the target population, spoke the relevant local languages, and received additional pre-interview training.

The sample size was estimated for the null hypothesis that there is no difference between M-FUND members and non-members in the proportion of one of the main outcomes namely the utilization of outpatient services during the previous six months. A two-sample proportions test was used to detect an odds ratio of 1·5 with a significance level set to 0·05 and a power of 80 %.

A sample of 408 cases was selected from the M-FUND database, consisting of individuals who were at least 18 years old and had been enrolled for a minimum of six months. The selection process involved picking every Nth member from the database to ensure that the estimated sample size was met. In addition, a total of 452 non-M-FUND migrant neighbors of the same sex and age (±5 years) were selected as controls. M-FUND interviewers recruited controls by asking for participants in the same neighborhood. Because there were multiple neighbors in most cases, interviewers were able to identify a non-member control. Only one person per household was selected. We excluded M-FUND members who were also members of another insurance scheme to avoid bias. In contrast, controls were enrolled regardless of their membership in other insurance schemes. We also excluded pregnant women and mothers who had given birth within the previous six months from the analysis because of the special needs that are covered by a special M-FUND pregnancy protection program, which require further study beyond the scope of this manuscript. The randomly selected M-FUND members were approached by M-FUND community workers. Participants received an allowance of THB 150 (≈ US$ 4·28), which was considered adequate given the local socio-economic context.

In addition to collecting socio-demographics including information on education, income and legal documents, the interview consisted of questions on health care utilization such as the frequency and types of outpatient, inpatient, and emergency care services used in the previous six months. The interview questions are given in Supplementary Table S14.

The study adhered to the Declaration of Helsinki and was approved by the ethics committees of the Medical Faculty of Heidelberg University, Germany, and of the Tak Provincial Public Health Office, Thailand.

### 2.2 Data analysis

After descriptive analysis, crude odds ratios with Chi² tests were calculated for M-FUND membership and independent variables to assess crude associations. To address research question (a), four multivariable logistic regression models were constructed to examine the association between membership status and (i) outpatient service utilization, (ii) inpatient service utilization, (iii) emergency care utilization, and (iv) no over-the-counter medicine purchase. Research question (b) was addressed by a subgroup analysis of participants who had seen a physician in the past six months. Here the following associations with membership status were modeled: (v) utilized any additional service, (vi) utilized x-ray/computed tomography scan or ultrasound, (vii) utilized blood test, (viii) utilized urine or another specimen test, (ix) utilized specialist consultation, (x) utilized treatment of an injury, (xi) utilized infusion/injection. All multivariable models were adjusted with the matching variables sex, age (<50 and ≥50 years), and district and with literacy, ethnicity, religion, marital status, legal document, educational attainment, years in Thailand, occupation, household income, and diagnosed with a chronic or communicable disease. Occupations were grouped into categories according to the degree of physical work (Supplementary Table S1). In models v-xi, the independent variables religion, ethnicity, and marital status were grouped into broader categories due to the small number of observations. All models were additionally disaggregated by sex if at least ten participants reported the respective outcome.

All analyses were complete case analyses and were performed using Stata 15.1 IC for Windows (StataCorp LLC, 4905 Lakeway Drive, College Station, TX 77845, USA), with *p* < 0·05 considered statistically significant.

### 2.3 Role of funding source

Dreamlopments Social Enterprise and Foundation contributed about 20 % € of the funding to cover data collection material and human resources costs. Representatives from the Social Enterprise were asked to give feedback during the development of the interview questionnaire and discussion of the results.

The German Academic Exchange Service provided about 40 % of the funding as a stipend for the data collection period, covering the living expenses and travel to country of research costs of the author AK and her family. The organization was not involved in the research.

## 3 Results

A total of 962 individuals were interviewed for this study. We excluded 102 individuals who were either M-FUND members with an additional health insurance scheme (*N* = 24) or were currently pregnant or within six months post giving birth (*N* = 78). The study sample consisted of 860 participants, 408 (47·4 %) M-FUND members (cases) and 452 (52·6 %) non-members (controls) (see Table 1). The non-members were mainly uninsured (88·1 %). The median age of non-members was 38 years and that of members 40 years, and in both groups about one quarter was over 50 years. 617 (71·7 %) participants were women and 243 (28·2 %) were men. The majority of participants lived in the Mae Sot district and stayed in Thailand for more than ten years (51·8 % of non-members and 62·3 % of M-FUND members). The ethnic groups reflected the geographical proximity to Myanmar, with the two main groups being the Bamar (49·3 % of non-members and 44·9 % of members) and the Karen (25·9 % of non-members and 27·9 % of members). The majority of participants were Buddhist (72·8 % of non-members and 69·9 % of members) and married (68·1 % of non-members and 75·0 % of members). Many had no legal documents (41·2 % of non-members and 44·9 % of members), or only informal papers issued by a local police officer or the village authority. With respect to education, the majority (68·2 % of non-members and 72·6 % of members) had completed at least primary school. Almost half of the participants were unemployed (42·7 % of non-members and 47·1 % of members), with more than 50 % of women being housewives. Among those who were employed, non-members worked more often in physically demanding jobs (37·0 %) than members (26·7 %). Household income ranged between 2000 and 8000 THB/month (≈US$ 58–233) for the majority of participants (61·7 % of non-members and 52·7 % of members). Overall, the completion of the survey was very high, resulting in few missing data of less than 1 %, except for household income (4·1 %).

**Table 1:** Descriptive data on covariables separated by insurance membership status.

Covariables	Non-members (*N* = 452)		M-FUND members (*N* = 408)	
	N	%	N	%
Age (median, range (years))	(38, 18–84)		(40, 18–88)	..
<50	341	75·4	303	74·3
≥50	111	24·6	105	25·7
Sex*				
Male	122	27·0	121	29·7
Female	330	73·0	287	70·3
District				
Mae Sot	381	84·3	347	85·1
Mae Ramat	47	10·4	41	10·1
Phop Phra	24	5·3	20	4·9
Duration in Thailand (years)				
0–5	107	23·7	64	15·7
6–10	111	24·6	90	22·1
>10	234	51·8	254	62·3
Ethnicity				
Bamar	223	49·3	183	44·9
Karen	117	25·9	114	27·9
Pa-O/Mon/Shan/Rakhine/other	112	24·8	111	27·2
Religion				
Buddhism	329	72·8	285	69·9
Islam	45	10·0	48	11·8
Christianity and other	78	17·3	75	18·4
Marital Status				
Single	74	16·4	56	13·7
Married	308	68·1	306	75·0
Divorced	28	6·2	12	2·9
Widowed	42	9·3	33	8·1
Legal document				
No legal document	186	41·2	183	44·9
Village authority or police paper	103	22·8	91	22·3
Formal legal document	163	36·1	134	32·8
Highest Education				
None	52	11·5	47	11·5
Part of primary	92	20·4	64	15·7
Primary	121	26·8	121	29·7
Secondary/high school/vocational school/university	187	41·4	175	42·9
Literacy				
No	66	14·6	54	12·2
Yes	386	85·4	354	86·8
Occupation				
Physically demanding jobs	167	37·0	109	26·7
Less physically demanding jobs	92	20·4	107	26·2
Unemployed	193	42·7	192	47·1
Household monthly Income (THB)				
0–2000	33	7·3	43	10·5
>2000–8000	279	61·7	215	52·7
>8000–12.000	104	23·0	96	23·5
>12.000	17	3·8	38	9·3
Don't know/no response (missing)	19	4·2	16	3·9
Need for care (medicine or physician visit)				
No	219	48·5	190	46·6
Yes	233	51·6	218	53·4
Chronic disease diagnosed				
No	380	84·1	330	80·9
Yes	72	15·9	78	19·1
Communicable disease diagnosed				
No	420	92·9	381	93·4
Yes	32	7·1	27	6·6

⁎ for all participants the reported gender was the same as the sex.

About 61 % of non-members and 70 % of M-FUND members did not buy over-the-counter medicines in the previous six months (see Table 2). Outpatient services, inpatient services, and emergency care was used by 26·8 %, 1·3 %, and 2·0 % of the non-members and by 37·3 %, 4·7 %, and 3·7 % of the members, respectively. Among those who visited a physician within the past six months (51·6 % of non-members; 53·4 % of members), 52·8 % of non-members and 69·7 % of members used a specific service during their visit. While vaccination and injury treatment (75 % men) services were rarely used, urine or specimen tests were almost equally used by both groups. Members utilized specialist consultations and infusions or injections somewhat more frequently than non-members. Larger differences were observed in the utilization of blood tests and X-ray or computed tomography scan or ultrasound procedures with frequencies of 17·2 % versus 28·4 % and 6 % versus 16·1 % for non-members and members, respectively.

**Table 2:** Descriptive data on outcomes separated by insurance membership status.

Outcomes	Non-members (*N* = 452)		M-FUND members (*N* = 408)	
	N	%	N	%
**Health care utilization in the last six months**				
Utilized outpatient services	121	26·8	152	37·3
Utilized inpatient services	6	1·3	19	4·7
Utilized emergency care	9	2·0	15	3·7
Bought no medicines over-the-counter	275	60·8	285	69·9
Types of services provided when visiting a physician*				
Utilized any service	123	52·8	152	69·7
Utilized vaccination	3	1·3	3	1·4
Utilized X-ray/ computed tomography scan or ultrasound	14	6·0	35	16·1
Utilized blood test	40	17·2	62	28·4
Utilized urine or another specimen test	22	9·4	18	8·3
Utilized specialist consultation	36	15·5	43	19·7
Utilized treatment for injury	5	2·2	11	5·1
Utilized infusion/injection	75	32·2	79	36·2

⁎ Restricted to participants who visited a physician.

A comparison of members and non-members with respect to their need for care defined as buying medicine or visiting a physician showed no difference (odds ratio 1·08, 95 % confidence interval 0·83–1·41). Other bivariable analyses showing the association between membership status and the socio-demographic variables are presented in the Supplementary Table S2. Among men, associations were only seen for household income and among women for duration in Thailand, marital status, occupation, and household income.

Results of the regression analyses of research question a) are presented in Table 3 (complete multivariable regression models in Tables S3–S6). M-FUND members had significantly higher odds of using outpatient services in comparison to non-members (adjusted odds ratio (OR) 1·74 (95 % confidence interval (CI) 1·24–2·44)) with men having a higher adjusted OR than women (men 2·34, 1·11–4·90; women 1·59, 1·08–2·35). Adjusted ORs for inpatient services, in particular among women, and for not having bought medicines over-the-counter were also significantly higher for members with 2.96 (1·11–7·92) and 1·67 (1·22–2·27), respectively. Further, odds for the utilization of emergency care were also elevated for members (1·89, 0·78–4·56) and particularly among men (3·01, 0·63–14·88), however not reaching statistical significance.

**Table 3:** Analysis of M-FUND membership and utilization of health services in the last six months (research question a).

		Men				Women				Total
		M-FUND member		OR	adj. OR†	M-FUND member		OR	adj. OR†	adj. OR†
research question (a)		yes	no			yes	no			
(i) Utilized outpatient services	yes	41	24	2·12*	2·34*	111	97	1·52*	1·59*	1·74*
	no	79	98			176	233			
(ii) Utilized inpatient services	yes	5	2	2·59	..	14	4	4·18*	..	2·96*
	no	116	120			273	326			
(iii) Utilized emergency care	yes	7	3	2·44	3·01	8	6	1·55	1·24	1·89
	no	114	119			279	324			
(iv) Bought no medicines over-the-counter	yes	86	80	1·30	1·59	199	195	1·59*	1·70*	1·67*
	no	34	41			86	134			

† Adjusted for demographic and socioeconomic variables shown in Table 1. (Detailed results see Supplementary Tables S3–S6).

⁎ *p* < 0·05.

Table 4 displays the results of research question b) (types of health services utilized during a physician visit; complete multivariable regression models in Tables S7–S13). Members had higher odds of utilizing any service during their last physician visit (2·31, 1·49–3·58) in comparison to non-members and the adjusted OR of men was higher than that of women. Utilization of X-ray, computed tomography scan or ultrasound showed also a higher adjusted OR for members of 2·62 (1·29–5·29). The adjusted OR for the utilization of blood tests appears to be higher, especially for male members (3·59, 0·98–13·20), however, not statistically significant. The utilization of urine or another specimen test, specialist consultation, treatment for injury, and infusions or injections showed no differences.

**Table 4:** Analysis of M-FUND membership and types of health services utilized among the participants who had seen a physician in the last six months (research question b).

		Men				Women				Total
		M-FUND member		OR	adj. OR†	M-FUND member		OR	adj. OR†	adj. OR†
research question (b)		yes	no			yes	no			
(v) Utilized any service	yes	46	27	3·30*	5·34*	106	96	1·75*	1·95*	2·31*
	no	15	29			51	81			
(vi) Utilized x-ray/ computed tomography scan or ultrasound	yes	10	3	3·46	3·12	25	11	2·86*	2·49*	2·62*
	no	51	53			132	166			
(vii) Utilized blood test	yes	21	9	2·74*	3·59*	41	31	1·67	1·39	1·64*
	no	40	47			116	146			
(viii) Utilized urine or another specimen test	yes	4	6	0·59	0·35	14	16	0·99	0·93	0·78
	no	57	50			143	161			
(ix) Utilized specialist consultation	yes	15	7	2·28	2·84	28	29	1·11	1·11	1·35
	no	46	49			129	148			
(x) Utilized treatment for injury	yes	8	4	1·96	..	3	1	3·43	..	2·36
	no	53	52			154	176			
(xi) Utilized infusion/injection	yes	19	12	1·66	1·56	60	63	1·12	1·22	1·32
	no	42	44			97	114			

† Adjusted for demographic and socioeconomic variables shown in Table 1. (Detailed results see Supplementary Tables S3–S6).

⁎ *p* < 0·05.

## 4 Discussion

Our findings indicate that migrants enrolled in the M-FUND use healthcare services more often compared to those who are not members. Additionally, M-FUND members exhibit increased usage of additional services during physician visits, likely attributable to financial barriers being lessened for both patients and providers.

As Thailand strives to achieve universal health coverage, health microinsurance such as the M-FUND can help bridge the gap, especially for undocumented and unemployed migrants. Many housewives and elderly migrants as well as daily workers are undocumented and are therefore ineligible for governmental insurance schemes. Consistent with the findings of an earlier qualitative case study and a study on maternal health, our data show that the M-FUND successfully enrolls this particularly vulnerable group of migrants (Tschirhart et al., 2021; Pudpong et al., 2019). By not requiring long-term upfront payments and official documents, the M-FUND fits into the reality of migrants' lives.

We observed few significant differences between men and women, but this may be due to the limited number of observations. However, differences in point estimates suggest sex-specific patterns of utilization. The higher utilization in men, of outpatient services, emergency care and using any specific service during a physician visit may be due to men being more likely to work in hazardous injurious physical jobs, while women are more likely to be housewives. Inpatient services may be slightly higher for women because of the patriarchal society in which these migrants live in. Women may not want or be allowed to leave the house without their husband's permission and may be reluctant to see a male doctor. As a result, they may not seek care until they are seriously ill and need inpatient services (Ashley-Norman et al., 2022).

The positive effects observed in this study on health care utilization among M-FUND members are in line with findings from other studies of migrants enrolled in governmental health insurance schemes in Thailand (Pudpong et al., 2019; Aung et al., 2009; Myo-Lin-Zaw and Hongkrailert, 2016; Hu, 2009). Studies of domestic migrants in China also found that having health insurance increases the likelihood of visiting a physician and receiving care, and that insurance schemes increase access to basic health services and essential medicines (Zhu et al., 2008; Zhang et al., 2020). A study of migrant workers in Qatar found an increase in the use of outpatient and inpatient services (Liu et al., 2020).

Yet our study had a number of shortcomings and limitations. Most importantly, data collection took place during the COVID-19 pandemic and faced intermittent access restrictions to certain areas, making recruitment difficult. As a result, the estimated sample size for M-FUND members (425 cases) was not fully achieved, and data had to be collected primarily in Mae Sot district. In order to continue data collection within the constrains, community workers first recruited participants in accessible communities and then moved into closed areas after they were reopened. In Mae Sot, participants live close to M-FUND's partner health care providers, which may have led to overestimation of use of services. On the other hand, the control group included more than ten percent of individuals with a governmental or other private health insurance, which may have biased our results towards the null. More generally, the limited number of interviews led to difficulties in analysing rare outcomes such as inpatient services and emergency care use, as well as sex-specific differences in utilization. However, we still believe that the results of these models provide interesting information.

Another effect of the pandemic restrictions was that some community workers and interviewers could not distinguish between people who refused to participate and those who could not be enrolled for other reasons, such as moving without reporting or changing telephone numbers, making it impossible to calculate an accurate response rate. Assuming that all non-participants refused to participate, the response rate would have been approximately 60 %, which is in the range of another study on migrants in Thailand (Pinna et al., 2020). The case-control design that we used has its limitations as it does not allow conclusions on causality, and case-control studies often struggle with collecting data from a meaningful control group. We decided to match controls to cases for the main sources of confounding. In general, we were successful in finding suitable controls in the neighborhood of cases.

M-FUND had a positive impact on the utilization of all major categories of health services among migrant workers and their dependents in Thailand. Health microinsurance appears to be a promising way to reduce barriers to health care utilization among migrants, particularly disadvantaged migrant workers and their families in this setting. This study of the impact of health microinsurance for migrants provides important information for health policy and program planners in LMICs with a similar labor migration context.

In a broader context, it must be stated that the world of the 21st century is a world of migration. Refugees, internally displaced persons, and residents affected by complex emergencies are challenged by severe health problems (Banunle and Apau, 2019), and many countries struggle how to achieve universal health coverage under miserable situations for these population groups (Heudtlass et al., 2016). While the last two groups were not covered by this study, our results indicate that health microinsurance has the potential to ease the lives of millions severely suffering. It is beyond the scope of this study to reduce the number of people living in unstable conditions, but we can recommend considering health microinsurance to ease the lives of those suffering from these conditions worldwide.

More research is needed on health microinsurance for migrants in different settings, as well as for other underserved groups and on sex-specific impact on health services utilization.

## Funding

The research was in part funded by Dreamlopments Social Enterprise and Foundation (ap proximately 20 %), the German Academic Exchange Service (40 %) and own funding (40 %).

## Data availability

Anonymized data will be available on request.

## Declaration of generative AI and AI-assisted technologies in the writing process

During the preparation of this work the authors used *DeepL Write* in order to improve scientific writing style. After using this tool, the authors reviewed and edited the content as needed and take full responsibility for the content of the publication.

## CRediT authorship contribution statement

**Andrea König:** Writing – review & editing, Writing – original draft, Visualization, Resources, Project administration, Methodology, Investigation, Funding acquisition, Formal analysis, Data curation, Conceptualization. **Jarntrah Sappayabanphot:** Writing – review & editing, Conceptualization. **Li Liang:** Writing – review & editing, Resources, Conceptualization. **Steffen Fleßa:** Supervision, Writing – review & editing. **Volker Winkler:** Writing – review & editing, Visualization, Validation, Supervision, Resources, Methodology, Funding acquisition, Formal analysis, Conceptualization.

## Declaration of competing interest

The authors declare that they have no known competing financial interests or personal relationships that could have appeared to influence the work reported in this paper.

## Appendix Supplementary materials

Image, application 1
