# Supplementary material for: The impact of the health microinsurance M-FUND on the utilization of health services among migrant workers and their dependents in Thailand: A case-control study
Source: J Migr Health. 2024 May 13;9:100236. doi: 10.1016/j.jmh.2024.100236 (PMC11127229; doi:10.1016/j.jmh.2024.100236)
Supplement: Supplementary file 1 [file mmc1.docx]

**Table S1 Overview of occupations grouped into more general occupational categories according to the degree of physical work for bivariable and multivariable analysis.**

| **Occupation Category** | **Occupations included** |
| --- | --- |
| Physically demanding jobs | Construction worker, farm/agricultural worker, daily wage general worker, factory worker, domestic worker |
| Less physically demanding jobs | Shop worker, self-employed, NGO/health worker, other occupations |
| Unemployed | No occupation |

**Table S2 Bivariable analysis of M-FUND membership and socio-demographic variables disaggregated by sex. Data are presented as frequencies, odds ratios with 95% confidence intervals and p-values.**

|  | **Men** | | | **Women** | | | **Total** | | |
| --- | --- | --- | --- | --- | --- | --- | --- | --- | --- |
| **Response Variable** | **N** | **OR**  **(95% CI)** | **p-value** | **N** | **OR**  **(95% CI)** | **p-value** | **N** | **OR**  **(95% CI)** | **p-value** |
| **District** |  |  | 0·72 |  |  | 0·95 |  |  | 0·95 |
| Mae Sot | 209 | 1 |  | 519 | 1 |  | 728 | 1 |  |
| Mae Ramat | 19 | 1·10  (0·43-2·82) |  | 69 | 0·93  (0·56-1·55) |  | 88 | 0·96  (0·61-1·49) |  |
| Phop Phra | 15 | 0·66  (0·23-1·92) |  | 29 | 1·07  (0·51-2·26) |  | 44 | 0·92  (0·50-1·69) |  |
| **Literacy** |  |  | 0·98 |  |  | 0·54 |  |  | 0·56 |
| No | 26 | 1 |  | 94 | 1 |  | 120 | 1 |  |
| Yes | 217 | 0·99  (0·44-2·24) |  | 523 | 1·15  (0·74-1·79) |  | 740 | 1·12  (0·76-1·65) |  |
| **Sex** |  |  | ·· |  |  | ·· |  |  | 0·39 |
| Male | ·· | ·· |  | ·· | ·· |  | 243 | 1 |  |
| Female | ·· | ·· |  | ·· | ·· |  | 617 | 0·88  (0·65-1·18) |  |
| **Age** |  |  | 0·97 |  |  | 0·68 |  |  | 0·69 |
| 18-49 | 177 | 1 |  | 467 | 1 |  | 644 | 1 |  |
| >49 | 66 | 1·01  (0·57-1·78) |  | 150 | 1·08  (0·75-1·56) |  | 216 | 1·07  (0·78- 1·45) |  |
| **Duration in Thailand** |  |  | 0·51 |  |  | <0·01 |  |  | <0·01 |
| 0-5 years | 39 | 1 |  | 132 | 1 |  | 171 | 1 |  |
| 6-10 years | 52 | 1·11  (0·48-2·56) |  | 149 | 1·44  (0·89-2·33) |  | 201 | 1·36  (0·89-2·06) |  |
| >10 years | 152 | 1·44  (0·71-2·91) |  | 336 | 1·94  (1·28-2·94) |  | 488 | 1·82  (1·27-2·59) |  |
| **Ethnicity** |  |  | 0·45 |  |  | 0·45 |  |  | 0·40 |
| Bamar | 108 | 1 |  | 318 | 1 |  | 406 | 1 |  |
| Karen | 76 | 1·52  (0·84-2·77) |  | 170 | 1·06  (0·72-1·56) |  | 231 | 1·19  (0·86-1·64) |  |
| Pa-O / Mon/ Shan/ Rakhine | 14 | 1·88  (0·63-5·67) |  | 48 | 0·83  (0·47-1·46) |  | 74 | 0·98  (0·60-1·61) |  |
| Other (mainly Muslim) | 49 | 1·31  (0·66-2·58) |  | 103 | 1·36  (0·86-2·14) |  | 149 | 1·34  (0·92-1·95) |  |
| **Religion** |  |  | 0·85 |  |  | 0·69 |  |  | 0·59 |
| Buddhism | 159 | 1 |  | 455 | 1 |  | 614 | 1 |  |
| Christianity or other religion | 35 | 1·23  (0·59-2·57) |  | 58 | 1·20  (0·69-2·07) |  | 93 | 1·23  (0·80-1·91) |  |
| Islam | 49 | 1·00  (0·53-1·89) |  | 104 | 1·15  (0·75-1·77) |  | 153 | 1·11  (0·78-1·58) |  |
| **Marital Status** |  |  | 0·77 |  |  | 0·02 |  |  | 0·06 |
| Single | 52 | 1·07  (0·57-1·99) |  | 78 | 0·60  (0·37-0·99) |  | 130 | 0·76  (0·52-1·12) |  |
| Married | 173 | 1 |  | 441 | 1 |  | 614 | 1 |  |
| Divorced | 9 | 0·79  (0·21-3·05) |  | 31 | 0·35  (0·15-0·81) |  | 40 | 0·43  (0·22-0·86) |  |
| Widowed | 9 | 0·49  (0·12-2·04) |  | 66 | 0·85  (0·50-1·42) |  | 75 | 0·79  (0·49-1·29) |  |
| **Legal document** |  |  | 0·10 |  |  | 0·14 |  |  | 0·51 |
| Undocumented | 77 | 1 |  | 292 | 1 |  | 369 | 1 |  |
| Village authority or police paper | 45 | 2·18  (1·02-4·64) |  | 149 | 0·69  (0·47-1·03) |  | 194 | 0·90  (0·63-1·27) |  |
| Formal legal document | 121 | 1·07  (0·60-1·90) |  | 176 | 0·76  (0·52-1·10) |  | 297 | 0·84  (0·62-1·14) |  |
| **Educational attainment** |  |  | 0·37 |  |  | 0·69 |  |  | 0·35 |
| None | 19 | 1 |  | 80 | 1 |  | 99 | 1 |  |
| Part of primary | 31 | 0·40  (0·12-1·29) |  | 125 | 0·90  (0·51-1·58) |  | 156 | 0·77  (0·46-1·28) |  |
| Primary/religious | 69 | 0·79  (0·28-2·21) |  | 173 | 1·18  (0·69-2·01) |  | 242 | 1·11  (0·69-1·77) |  |
| Secondary/high school/vocational school/university | 123 | 0·74  (0·28-1·96) |  | 239 | 1·10  (0·66-1·82) |  | 362 | 1·04  (0·66-1·62) |  |
| **Occupation** |  |  | 0·23 |  |  | 0·01 |  |  | <0·01 |
| Physically demanding jobs | 109 | 1 |  | 167 | 1 |  | 276 | 1 |  |
| Less physically demanding jobs | 80 | 1·41  (0·79-2·51) |  | 119 | 2·09  (1·30-3·38) |  | 199 | 1·78  (1·23-2·58) |  |
| Unemployed | 54 | 1·71  (0·89-3·31) |  | 331 | 1·65  (1·12-2·41) |  | 385 | 1·52  (1·11-2·09) |  |
| **Household income** |  |  | 0·04 |  |  | 0·05 |  |  | <0·01 |
| 0-2000 THB | 26 | 1 |  | 50 | 1 |  | 76 | 1 |  |
| 2001-8000 THB | 146 | 0·43  (0·18-1·02) |  | 348 | 0·70  (0·39-1·27) |  | 494 | 0·59  (0·36- 0·96) |  |
| 8001-12·000 THB | 50 | 0·53  (0·20-1·41) |  | 150 | 0·83  (0·44-1·58) |  | 200 | 0·71  (0·42- 1·21) |  |
| 12·001-30·000 THB | 17 | 1·72  (0·43-6·85) |  | 38 | 1·78  (0·74-4·34) |  | 55 | 1·72  (0·83- 3·56) |  |
| **Need for care (medicine or physician visit)** |  |  | 0·48 |  |  | 0·79 |  |  | 0·58 |
| No | 126 | 1 |  | 283 | 1 |  | 409 | 1 |  |
| Yes | 117 | 1·20 (0·72-1·98) |  | 334 | 1·04 (0·76-1·43) |  | 451 | 1·08 (0·83-1·41) |  |
| **Chronic disease diagnosed** |  |  | 0·17 |  |  | 0·46 |  |  | 0·22 |
| No | 212 | 1 |  | 498 | 1 |  | 710 | 1 |  |
| Yes | 31 | 1·71  (0·79-3·69) |  | 119 | 1·17  (0·78-1·74) |  | 150 | 1·25  (0·88-1·78) |  |
| **Non-Chronic disease diagnosed** |  |  | 0·80 |  |  | 0·88 |  |  | 0·79 |
| No | 228 | 1 |  | 573 | 1 |  | 801 | 1 |  |
| Yes | 15 | 0·88  (0·31-2·49) |  | 44 | 0·96  (0·52-1·77) |  | 59 | 0·93 (0·55-1·58) |  |

**Table S3 Model i) No over the counter medicine purchases: Multivariable analysis of M-FUND membership and socio-demographic variables disaggregated by sex.**

|  | **Men** | | **Women** | | **Total** | |
| --- | --- | --- | --- | --- | --- | --- |
| **M-FUND membership** | **OR**  **(95% CI)** | **p-value** | **OR**  **(95% CI)** | **p-value** | **OR**  **(95% CI)** | **p-value** |
| **Bought no medication over the counter** |  | 0·17 |  | 0·01 |  | <0·01 |
| No | 1 |  | 1 |  | 1 |  |
| Yes | 1·59  (0·82-3·03) |  | 1·70  (1·16-2·44) |  | 1·67  (1·22-2·27) |  |
| **District name** |  | 0·85 |  | 0·62 |  | 0·72 |
| Mae Ramat | 1 |  | 1 |  | 1 |  |
| Mae Sot | 0·72  (0·23-2·29) |  | 0·87  (0·47-1·60) |  | 0·85  (0·50- 1·42) |  |
| Phop Phra | 0·81  (0·16-4·14) |  | 1·28  (0·49-3·32) |  | 1·04  (·47-2·31) |  |
| **Age** |  | 0·41 |  | 0·69 |  | 0·50 |
| 18-49 | 1 |  | 1 |  | 1 |  |
| >49 | 0·72  (0·33-1·58) |  | 0·91  (0·56-1·48) |  | 0·87  (0·59-1·30) |  |
| **Sex** |  | ·· |  | ·· |  | 0·37 |
| Male | ·· |  | ·· |  | 1 |  |
| Female | ·· |  | ·· |  | 0·85  (0·60-2·21) |  |
| **Literacy** |  | 0·37 |  | 0·34 |  | 0·20 |
| No | 1 |  | 1 |  | 1 |  |
| Yes | 1·90  (0·47-7·60) |  | 1·76  (0·56-5·59) |  | 1·73  (0·75-4·00) |  |
| **Ethnicity** |  | 0·72 |  | 0·47 |  | 0·56 |
| Bamar | 1 |  | 1 |  | 1 |  |
| Karen | 1·12  (0·45-2·80) |  | 0·85  (0·51-1·42) |  | 0·99  (0·65- 1·53) |  |
| Pa-O / Mon/ Shan/ Rakhine | 1·80  (0·50-6·54) |  | 0·79  (0·41-1·53) |  | 0·95  (0·54-1·66) |  |
| Other (mainly Muslim) | 2·25  (0·29-17·20) |  | 1·97  (0·62-6·24) |  | 1·98  (0·74- 5·31) |  |
| **Religion** |  | 0·79 |  | 0·50 |  | 0·45 |
| Buddhism | 1 |  | 1 |  | 1 |  |
| Christianity or other religion | 1·21  (0·39-3·76) |  | 1·21  (0·60-2·46) |  | 1·18  (0·66- 2·11) |  |
| Islam | 0·60  (0·08-4·62) |  | 0·58  (0·19-1·81) |  | 0·61  (0·23- 1·61) |  |
| **Marital status** |  | 0·36 |  | 0·03 |  | 0·02 |
| Single | 1 |  | 1 |  | 1 |  |
| Married | 1·33  (0·60-2·95) |  | 1·96  (1·09-3·53) |  | 1·57  (1·00- 2·47) |  |
| Divorced | 0·76  (0·14-4·11) |  | 0·71  (0·25-2·05) |  | 0·65  (0·28-1·54) |  |
| Widowed | 0·30  (0·04-1·97) |  | 1·45  (0·62-3·41) |  | 1·04  (0·50- 2·16) |  |
| **Legal document** |  | 0·06 |  | 0·50 |  | 0·31 |
| Undocumented | 1 |  | 1 |  | 1 |  |
| Village authority or police paper | 2·33  (0·96-5·65) |  | 0·83  (0·52-1·31) |  | 1·07  (0·72- 1·59) |  |
| Formal legal document | 0·90  (0·45-1·83) |  | 0·79  (0·51-1·22) |  | 0·80  (0·56- 1·14) |  |
| **Educational attainment** |  | 0·48 |  | 0·84 |  | 0·44 |
| None | 1 |  | 1 |  | 1 |  |
| Part of primary | 0·30  (0·07-1·41) |  | 0·57  (0·17-1·94) |  | 0·51  (0·21- 1·25) |  |
| Primary/religious | 0·48  (0·10-2·43) |  | 0·63  (0·18-2·23) |  | 0·64  (0·25- 1·63) |  |
| Secondary/high school/vocational school/university | 0·44  (0·08-2·35) |  | 0·62  (0·17-2·21) |  | 0·60  (0·23- 1·55) |  |
| **Chronic disease diagnosed** |  | 0·17 |  | 0·54 |  | 0·17 |
| No | 1 |  | 1 |  | 1 |  |
| Yes | 2·01  (0·75-5·41) |  | 1·16  (0·72-1·85) |  | 1·33  (0·88-2·01) |  |
| **Non-Chronic disease diagnosed** |  | 0·84 |  | 0·94 |  | 0·73 |
| No | 1 |  | 1 |  | 1 |  |
| Yes | 0·88  (0·25-3·04) |  | 0·98  (0·51-1·88) |  | 0·90  (0·51- 1·59) |  |
| **Duration in Thailand** |  | 0·34 |  | 0·01 |  | <0·01 |
| 0-5 years | 1 |  | 1 |  | 1 |  |
| 6-10 years | 0·98  (0·38-2·54) |  | 1·55  (0·92-2·60) |  | 1·45  (0·92-2·26) |  |
| >10 years | 1·56  (0·69-3·51) |  | 2·12  (1·32-3·40) |  | 2·00  (1·34-2·99) |  |
| **Occupation** |  | 0·18 |  | 0·06 |  | 0·06 |
| Physically demanding jobs | 1 |  | 1 |  | 1 |  |
| Less physically demanding jobs | 1·48  (0·73-2·99) |  | 1·96  (1·12-3·45) |  | 1·65  (1·08- 2·51) |  |
| Unemployed | 2·44  (0·92-6·47) |  | 1·32  (0·84-2·09) |  | 1·38  (0·94- 2·03) |  |
| **Household income** |  | 0·06 |  | 0·02 |  | <0·01 |
| 0-2000 THB | 1 |  | 1 |  | 1 |  |
| 2001-8000 THB | 0·74  (0·25-2·19) |  | 0·55  (0·28-1·09) |  | 0·56  (0·32-0·97) |  |
| 8001-12·000 THB | 1·05  (0·31-3·55) |  | 0·66  (0·32-1·38) |  | 0·73  (0·40- 1·34) |  |
| 12·001-30·000 THB | 4·53  (0·88-23·32) |  | 1·62  (0·62-4·25) |  | 1·98  (0·89- 4·44) |  |

**Table S4 Model ii) Outpatient care utilization: Multivariable analysis of M-FUND membership and socio-demographic variables disaggregated by sex.**

|  | **Men** | | **Women** | | **Total** | |
| --- | --- | --- | --- | --- | --- | --- |
| **M-FUND membership** | **OR**  **(95% CI)** | **p-value** | **OR**  **(95% CI)** | **p-value** | **OR**  **(95% CI)** | **p-value** |
| **Utilized outpatient care** |  | 0·03 |  | 0·02 |  | <0·01 |
| No | 1 |  | 1 |  | 1 |  |
| Yes | 2·34  (1·11-4·90) |  | 1·59  (1·08- 2·35) |  | 1·74  (1·24-2·44) |  |
| **District name** |  | 0·83 |  | 0·86 |  | 0·92 |
| Mae Ramat | 1 |  | 1 |  | 1 |  |
| Mae Sot | 0·73  (0·23-2·30) |  | 0·93  (0·51-1·69) |  | 0·90  (0·54-1·51) |  |
| Phop Phra | 0·63  (0·12-3·18) |  | 1·14  (0·45-2·91) |  | 0·89  (0·41-1·97) |  |
| **Age** |  | 0·19 |  | 0·49 |  | 0·23 |
| 18-49 | 1 |  | 1 |  | 1 |  |
| >49 | 0·59  (0·27-1·31) |  | 0·84  (0·52- 1·37) |  | 0·78  (0·53-1·17) |  |
| **Sex** |  | ·· |  | ·· |  | 0·26 |
| Male | ·· |  | ·· |  | 1 |  |
| Female | ·· |  | ·· |  | 0·82  (0·58- 1·16) |  |
| **Literacy** |  | 0·46 |  | 0·22 |  | 0·16 |
| No | 1 |  | 1 |  | 1 |  |
| Yes | 1·69  (0·43-6·71) |  | 2·04  (0·65-6·42) |  | 1·84  (0·79-4·25) |  |
| **Ethnicity** |  | 0·37 |  | 0·55 |  | 0·47 |
| Bamar | 1 |  | 1 |  | 1 |  |
| Karen | 1·33  (0·52-3·36) |  | 0·92  (0·55-1·54) |  | 1·10  (0·72-1·69) |  |
| Pa-O / Mon/ Shan/ Rakhine | 2·47  (0·70-8·64) |  | 0·87  (0·45-1·68) |  | 1·11  (0·64- 1·94) |  |
| Other (mainly Muslim) | 3·47  (0·43-27·97) |  | 2·06  (0·66-6·50) |  | 2·23  (0·83- 6·00) |  |
| **Religion** |  | 0·60 |  | 0·40 |  | 0·27 |
| Buddhism | 1 |  | 1 |  | 1 |  |
| Christianity or other religion | 1·19  (0·38-3·70) |  | 1·26  (0·62-2·56) |  | 1·22  (0·68- 2·19) |  |
| Islam | 0·40  (0·05-3·20) |  | 0·54  (0·17-1·67) |  | 0·53  (0·20- 1·40) |  |
| **Marital status** |  | 0·42 |  | 0·02 |  | 0·02 |
| Single | 1 |  | 1 |  | 1 |  |
| Married | 1·37  (0·62-3·02) |  | 1·87  (1·04-3·35) |  | 1·54  (0·98- 2·41) |  |
| Divorced | 0·91  (0·16-5·03) |  | ·63  (0·22-1·79) |  | 0·64  (0·27- 1·51) |  |
| Widowed | 0·34  (0·05-2·22) |  | 1·34  (0·57-3·14) |  | 1·00  (0·48- 2·08) |  |
| **Legal document** |  | 0·15 |  | 0·36 |  | 0·21 |
| Undocumented | 1 |  | 1 |  | 1 |  |
| Village authority or police paper | 1·85  (0·77-4·42) |  | 0·80  (0·51-1·25) |  | 0·98  (0·67-1·45) |  |
| Formal legal document | 0·82  (0·40-1·67) |  | 0·74  (0·48-1·15) |  | 0·74  (0·51-1·06) |  |
| **Educational attainment** |  | 0·38 |  | 0·72 |  | 0·32 |
| None | 1 |  | 1 |  | 1 |  |
| Part of primary | 0·32  (0·07-1·53) |  | 0·50  (0·15-1·69) |  | 0·49  (0·20-1·20) |  |
| Primary/religious | 0·68  (0·14-3·38) |  | 0·57  (0·16-1·98) |  | 0·65  (0·26-1·66) |  |
| Secondary/high school/vocational school/university | 0·60  (0·11-3·17) |  | 0·55  (0·15-1·96) |  | 0·60  (0·23-1·57) |  |
| **Chronic disease diagnosed** |  | 0·57 |  | 0·77 |  | 0·88 |
| No | 1 |  | 1 |  | 1 |  |
| Yes | 1·36  (0·47-3·93) |  | 0·93  (0·58-1·50) |  | 1·03  (0·68-1·58) |  |
| **Non-Chronic disease diagnosed** |  | 0·63 |  | 0·67 |  | 0·43 |
| No | 1 |  | 1 |  | 1 |  |
| Yes | 0·74  (0·22-2·51) |  | 0·87  (0·45-1·67) |  | 0·80  (0·45- 1·40) |  |
| **Duration in Thailand** |  | 0·44 |  | 0·01 |  | 0·01 |
| 0-5 years | 1 |  | 1 |  | 1 |  |
| 6-10 years | 0·84  (0·33-2·15) |  | 1·55  (0·92-2·60) |  | 1·39  (0·89-2·16) |  |
| >10 years | 1·33  (0·59-2·98) |  | 2·04  (1·27-3·27) |  | 1·87  (1·26- 2·80) |  |
| **Occupation** |  | 0·22 |  | 0·04 |  | 0·06 |
| Physically demanding jobs | 1 |  | 1 |  | 1 |  |
| Less physically demanding jobs | 1·44  (0·71-2·92) |  | 2·08  (1·19-3·66) |  | 1·67  (1·09- 2·54) |  |
| Unemployed | 2·33  (0·87-6·26) |  | 1·32  (0·84-2·08) |  | 1·34  (0·91-1·96) |  |
| **Household income** |  | 0·11 |  | 0·03 |  | <0·01 |
| 0-2000 THB | 1 |  | 1 |  | 1 |  |
| 2001-8000 THB | 0·70  (0·23-2·10) |  | 0·56  (0·29-1·11) |  | 0·55  (0·32-0·96) |  |
| 8001-12·000 THB | 1·07 (0·31-3·64) |  | 0·69  (0·33-1·41) |  | 0·74  (0·41- 1·34) |  |
| 12·001-30·000 THB | 3·45  (0·67-17·78) |  | 1·55  (0·60- 4·02) |  | 1·74  (0·79- 3·87) |  |

**Table S5 Model iii) Inpatient care utilization: Multivariable analysis of M-FUND membership and socio-demographic variables disaggregated by sex.**

|  | **Men** | | **Women** | | **Total** | |
| --- | --- | --- | --- | --- | --- | --- |
| **M-FUND membership** | **OR**  **(95% CI)** | **p-value** | **OR**  **(95% CI)** | **p-value** | **OR**  **(95% CI)** | **p-value** |
| **Utilized inpatient care** |  | 0·67 |  | 0·10 |  | 0·03 |
| No | 1 |  | 1 |  | 1 |  |
| Yes | 1·53  (0·21-11·06) |  | 2·80  (0·83-9·39) |  | 2·96  (1·11-7·92) |  |
| **District name** |  | 0·87 |  | 0·84 |  | 0·89 |
| Mae Ramat | 1 |  | 1 |  | 1 |  |
| Mae Sot | 0·74  (0·24-2·34) |  | 0·92  (0·51-1·68) |  | 0·88  (0·53-1·47) |  |
| Phop Phra | 0·71  (0·14-3·56) |  | 1·16  (0·45-2·97) |  | 0·93  (0·42-2·03) |  |
| **Age** |  | 0·29 |  | 0·55 |  | 0·36 |
| 18-49 | 1 |  | 1 |  | 1 |  |
| >49 | 0·66  (0·30-1·43) |  | 0·86  (0·53-1·41) |  | 0·83  (0·56-1·23) |  |
| **Sex** |  | ·· |  | ·· |  | 0·33 |
| Male | ·· |  | ·· |  | 1 |  |
| Female | ·· |  | ·· |  | 0·84  (0·60-1·19) |  |
| **Literacy** |  | 0·37 |  | 0·28 |  | 0·15 |
| No | 1 |  | 1 |  | 1 |  |
| Yes | 1·89  (0·47-7·53) |  | 1·88  (0·60-5·82) |  | 1·84  (0·80-4·26) |  |
| **Ethnicity** |  | 0·53 |  | 0·49 |  | 0·55 |
| Bamar | 1 |  | 1 |  | 1 |  |
| Karen | 1·18  (0·47-2·95) |  | 0·90  (0·54-1·50) |  | 1·04  (0·68-1·60) |  |
| Pa-O / Mon/ Shan/ Rakhine | 2·29  (0·65-8·09) |  | 0·82  (0·43-1·57) |  | 1·03  (0·59-1·80) |  |
| Other (mainly Muslim) | 2·29  (0·30-17·31) |  | 2·07  (0·65-6·58) |  | 2·07  (0·77-5·55) |  |
| **Religion** |  | 0·77 |  | 0·39 |  | 0·32 |
| Buddhism | 1 |  | 1 |  | 1 |  |
| Christianity or other religion | 1·20  (0·38-3·77) |  | 1·22  (0·60-2·47) |  | 1·21  (0·68-2·16) |  |
| Islam | 0·57  (0·07-4·34) |  | 0·51  (0·16-1·61) |  | 0·55  (0·21-1·47) |  |
| **Marital status** |  | 0·40 |  | 0·04 |  | 0·03 |
| Single | 1 |  | 1 |  | 1 |  |
| Married | 1·38  (0·63-3·05) |  | 1·83  (1·02-3·28) |  | 1·53  (0·97-2·40) |  |
| Divorced | 0·84  (0·15-4·58) |  | 0·65  (0·23-1·86) |  | 0·64  (0·27-1·50) |  |
| Widowed | 0·34  (0·05-2·24) |  | 1·39  (0·60-3·25) |  | 1·04  (0·50-2·16) |  |
| **Legal document** |  | 0·09 |  | 0·43 |  | 0·27 |
| Undocumented | 1 |  | 1 |  | 1 |  |
| Village authority or police paper | 2·04  (0·85-4·88) |  | 0·81  (0·52-1·27) |  | 1·00  (0·68-1·47) |  |
| Formal legal document | 0·83  (0·41-1·67) |  | 0·77  (0·50-1·19) |  | 0·76  (0·53-1·09) |  |
| **Educational attainment** |  | 0·41 |  | 0·74 |  | 0·31 |
| None | 1 |  | 1 |  | 1 |  |
| Part of primary | 0·30  (0·06-1·41) |  | 0·52  (0·16-1·71) |  | 0·46  (0·19-1·14) |  |
| Primary/religious | 0·55  (0·11-2·71) |  | 0·58  (0·17-2·00) |  | 0·61  (0·24-1·54) |  |
| Secondary/high school/vocational school/university | 0·49  (0·09-2·58) |  | 0·56  (0·16-1·98) |  | 0·56  (0·22-1·45) |  |
| **Chronic disease diagnosed** |  | 0·18 |  | 0·90 |  | 0·43 |
| No | 1 |  | 1 |  | 1 |  |
| Yes | 1·97  (0·73-5·33) |  | 1·03  (0·65-1·64) |  | 1·18  (0·78-1·78) |  |
| **Non-Chronic disease diagnosed** |  | 0·64 |  | 0·72 |  | 0·41 |
| No | 1 |  | 1 |  | 1 |  |
| Yes | 0·75  (0·35-2·35) |  | 0·89  (0·46-1·71) |  | 0·79  (0·45- 1·39) |  |
| **Duration in Thailand** |  | 0·44 |  | 0·01 |  | 0·01 |
| 0-5 years | 1 |  | 1 |  | 1 |  |
| 6-10 years | 0·91  (0·21-2·60) |  | 1·51  (0·90-2·53) |  | 1·40  (0·90-2·18) |  |
| >10 years | 1·41  (0·63-3·13) |  | 2·03  (1·27-3·24) |  | 1·88  (1·26- 2·80) |  |
| **Occupation** |  | 0·23 |  | 0·06 |  | 0·07 |
| Physically demanding jobs | 1 |  | 1 |  | 1 |  |
| Less physically demanding jobs | 1·48  (0·74-2·98) |  | 1·97  (1·12-3·44) |  | 1·62  (1·07- 2·47) |  |
| Unemployed | 2·28  (0·85-6·11) |  | 1·32  (0·84-2·08) |  | 1·33  (0·90-1·94) |  |
| **Household income** |  | 0·07 |  | 0·03 |  | <0·01 |
| 0-2000 THB | 1 |  | 1 |  | 1 |  |
| 2001-8000 THB | 0·67  (0·23-1·98) |  | 0·60  (0·31-1·19) |  | 0·58  (0·33- 1·01) |  |
| 8001-12·000 THB | 0·95  (0·28-3·17) |  | 0·73  (0·35-1·50) |  | 0·76  (0·42-1·38) |  |
| 12·001-30·000 THB | 3·74  (0·74-18·87) |  | 1·72  (0·66-4·45) |  | 1·96  (0·88-4·36) |  |

**Table S6 Model iv) Emergency care utilization: Multivariable analysis of M-FUND membership and socio-demographic variables disaggregated by sex.**

|  | **Men** | | **Women** | | **Total** | |
| --- | --- | --- | --- | --- | --- | --- |
| **M-FUND membership** | **OR**  **(95% CI)** | **p-value** | **OR**  **(95% CI)** | **p-value** | **OR**  **(95% CI)** | **p-value** |
| **Utilized emergency care** |  | 0·17 |  | 0·72 |  | 0·16 |
| No | 1 |  | 1 |  | 1 |  |
| Yes | 3·01  (0·63-14·38) |  | 1·24  (0·39-4·00) |  | 1·89  (0·78-4·56) |  |
| **District name** |  | 0·94 |  | 0·85 |  | 0·90 |
| Mae Ramat | 1 |  | 1 |  | 1 |  |
| Mae Sot | 0·82  (0·26-2·57) |  | 0·91  (0·50-1·66) |  | 0·89  (0·53-1·49) |  |
| Phop Phra | 0·83  (0·16-4·22) |  | 1·13  (0·44-2·89) |  | 0·94  (0·43-2·05) |  |
| **Age** |  | 0·29 |  | 0·63 |  | 0·34 |
| 18-49 | 1 |  | 1 |  | 1 |  |
| >49 | 0·66  (0·30-1·43) |  | 0·89  (0·55-1·44) |  | 0·83  (0·56-1·22) |  |
| **Sex** |  | ·· |  | ·· |  | 0·34 |
| Male | ·· |  | ·· |  | 1 |  |
| Female | ·· |  | ·· |  | 0·85  (0·60-1·19) |  |
| **Literacy** |  | 0·37 |  | 0·25 |  | 0·15 |
| No | 1 |  | 1 |  | 1 |  |
| Yes | 1·90  (0·46-7·79) |  | 1·94  (0·62-6·01) |  | 1·84  (0·80-4·25) |  |
| **Ethnicity** |  | 0·45 |  | 0·51 |  | 0·52 |
| Bamar | 1 |  | 1 |  | 1 |  |
| Karen | 1·33  (0·52-3·35) |  | 0·90  (0·54-1·49) |  | 1·07  (0·70-1·63) |  |
| Pa-O / Mon/ Shan/ Rakhine | 2·28  (0·65-8·08) |  | 0·80  (0·42-1·54) |  | 1·02  (0·58-1·77) |  |
| Other (mainly Muslim) | 3·12  (0·38-25·48) |  | 1·97  (0·62-6·21) |  | 2·12  (0·79-5·69) |  |
| **Religion** |  | 0·69 |  | 0·45 |  | 0·34 |
| Buddhism | 1 |  | 1 |  | 1 |  |
| Christianity or other religion | 1·08  (0·35-3·37) |  | 1·21  (0·60-2·45) |  | 1·18  (0·66-2·10) |  |
| Islam | 0·43  (0·05-3·51) |  | 0·55  (0·18-1·72) |  | 0·55  (0·21-1·45) |  |
| **Marital status** |  | 0·42 |  | 0·03 |  | 0·03 |
| Single | 1 |  | 1 |  | 1 |  |
| Married | 1·37  (0·62-3·03) |  | 1·87  (1·04-3·35) |  | 1·54  (0·98-2·42) |  |
| Divorced | 0·88  (0·16-4·76) |  | 0·64  (0·23-1·83) |  | 0·66  (0·28-1·54) |  |
| Widowed | 0·34  (0·05-2·23) |  | 1·39  (0·60-3·25) |  | 1·05  (0·51-2·18) |  |
| **Legal document** |  | 0·11 |  | 0·45 |  | 0·28 |
| Undocumented | 1 |  | 1 |  | 1 |  |
| Village authority or police paper | 1·95  (0·82-4·65) |  | 0·81  (0·52-1·27) |  | 1·02  (0·69-1·49) |  |
| Formal legal document | 0·82  (0·40-1·65) |  | 0·78  (0·50-1·20) |  | 0·77  (0·54-1·10) |  |
| **Educational attainment** |  | 0·36 |  | 0·74 |  | 0·34 |
| None | 1 |  | 1 |  | 1 |  |
| Part of primary | 0·30  (0·06-1·42) |  | 0·52  (0·16-1·72) |  | 0·48  (0·20-1·19) |  |
| Primary/religious | 0·60  (0·12-3·05) |  | 0·58  (0·17-2·01) |  | 0·63  (0·25-1·60) |  |
| Secondary/high school/vocational school/university | 0·54  (0·10-2·91) |  | 0·56  (0·16-1·96) |  | 0·58  (0·23-1·50) |  |
| **Chronic disease diagnosed** |  | 0·15 |  | 0·81 |  | 0·36 |
| No | 1 |  | 1 |  | 1 |  |
| Yes | 2·07  (0·77-5·58) |  | 1·06  (0·67-1·68) |  | 1·21  (0·81-1·82) |  |
| **Non-Chronic disease diagnosed** |  | 0·61 |  | 0·75 |  | 0·48 |
| No | 1 |  | 1 |  | 1 |  |
| Yes | 0·72  (0·21-2·52) |  | 0·90  (0·47-1·72) |  | 0·82  (0·47-1·43) |  |
| **Duration in Thailand** |  | 0·47 |  | 0·01 |  | 0·01 |
| 0-5 years | 1 |  | 1 |  | 1 |  |
| 6-10 years | 0·88  (0·34-2·25) |  | 1·56  (0·93-2·61) |  | 1·42  (0·91-2·21) |  |
| >10 years | 1·35  (0·61-3·00) |  | 2·06  (1·29-3·29) |  | 1·91  (1·28-2·84) |  |
| **Occupation** |  | 0·21 |  | 0·05 |  | 0·06 |
| Physically demanding jobs | 1 |  | 1 |  | 1 |  |
| Less physically demanding jobs | 1·56  (0·77-3·15) |  | 1·99  (1·14-3·48) |  | 1·65  (1·08-2·51) |  |
| Unemployed | 2·25  (0·85-5·99) |  | 1·31  (0·84-2·06) |  | 1·35  (0·92-1·98) |  |
| **Household income** |  | 0·06 |  | 0·03 |  | <0·01 |
| 0-2000 THB | 1 |  | 1 |  | 1 |  |
| 2001-8000 THB | 0·65  (0·22-1·93) |  | 0·58  (0·30-1·14) |  | 0·56  (0·33-0·98) |  |
| 8001-12·000 THB | 0·93  (0·28-3·12) |  | 0·70  (0·34-1·43) |  | 0·74  (0·41-1·35) |  |
| 12·001-30·000 THB | 3·70  (0·73-18·77) |  | 1·63  (0·63-4·23) |  | 1·90  (0·86-4·22) |  |

**Table S7 Model v) Utilized any service: Multivariable analysis of M-FUND membership and socio-demographic variables disaggregated by sex.**

|  | **Men** | | **Women** | | **Total** | |
| --- | --- | --- | --- | --- | --- | --- |
| **M-FUND membership** | **OR**  **(95% CI)** | **p-value** | **OR**  **(95% CI)** | **p-value** | **OR**  **(95% CI)** | **p-value** |
| **Utilized any service** |  | <0·01 |  | 0·01 |  | <0·01 |
| No | 1 |  | 1 |  | 1 |  |
| Yes | 5·34  (1·83-15·60) |  | 1·95  (1·17-3·27) |  | 2·31  (1·49-3·58) |  |
| **District name** |  | 0·90 |  | 0·61 |  | 0·89 |
| Mae Ramat | 1 |  | 1 |  | 1 |  |
| Mae Sot | 1·45  (0·19-11·35) |  | 1·03  (0·45-2·40) |  | 1·06  (0·51-2·22) |  |
| Phop Phra | 1·06  (0·09-12·64) |  | 1·63  (0·52-5·11) |  | 1·28  (0·48-3·45) |  |
| **Age** |  | 0·74 |  | 0·87 |  | 0·77 |
| 18-49 | 1 |  | 1 |  | 1 |  |
| >49 | 1·23  (0·37-4·04) |  | 0·95  (0·51-1·77) |  | 0·95  (0·57-1·59) |  |
| **Sex** |  | ·· |  | ·· |  | 0·32 |
| Male | ·· |  | ·· |  | 1 |  |
| Female | ·· |  | ·· |  | 0·79  (0·48-1·29) |  |
| **Literacy** |  | 0·11 |  | 0·19 |  | 0·05 |
| No | 1 |  | 1 |  | 1 |  |
| Yes | 5·23  (0·67-40·60) |  | 2·66  (0·61-11·57) |  | 3·29  (1·05-10·31) |  |
| **Ethnicity** |  | 0·48 |  | 0·67 |  | 0·49 |
| Bamar | 1 |  | 1 |  | 1 |  |
| Karen | 1·50  (0·39-5·80) |  | 0·78  (0·37-1·64) |  | 0·91  (0·49-1·68) |  |
| Other | 2·93  (0·52-16·62) |  | 1·13  (0·51-2·51) |  | 1·36  (0·69-2·68) |  |
| **Religion** |  | 0·17 |  | 0·86 |  | 0·55 |
| Buddhism | 1 |  | 1 |  | 1 |  |
| Other religion | 0·33  (0·07-1·62) |  | 1·07  (0·49-2·35) |  | 0·79  (0·41-1·54) |  |
| **Marital status** |  | 0·21 |  | 0·19 |  | 0·18 |
| Single | 1 |  | 1 |  | 1 |  |
| Married | 2·70  (0·71-10·25) |  | 0·86  (0·34-2·16) |  | 1·40  (0·69-2·84) |  |
| Divorced or widowed | 0·71  (0·06-8·61) |  | 0·47  (0·16-1·42) |  | 0·74  (0·30-1·88) |  |
| **Legal document** |  | 0·17 |  | 0·34 |  | 0·07 |
| Undocumented | 1 |  | 1 |  | 1 |  |
| Village authority or police paper | 1·38  (0·32-5·96) |  | 1·04  (0·56-1·94) |  | 1·21  (0·70-2·10) |  |
| Formal legal document | 0·43  (0·14-1·36) |  | 0·66  (0·36-1·22) |  | 0·63  (0·38-1·05) |  |
| **Educational attainment** |  | 0·04 |  | 0·33 |  | 0·05 |
| None | 1 |  | 1 |  | 1 |  |
| Part of primary | 0·07  (0·01-0·66) |  | 0·24  (0·05-1·15) |  | 0·19  (0·06-0·63) |  |
| Primary/religious | 0·36  (0·04-3·42) |  | 0·23  (0·04-1·22) |  | 0·25  (0·07-0·89) |  |
| Secondary/high school/vocational school/university | 0·41  (0·04-3·89) |  | 0·21  (0·04-1·13) |  | 0·25  (0·07-0·90) |  |
| **Chronic disease diagnosed** |  | 0·97 |  | 0·81 |  | 0·80 |
| No | 1 |  | 1 |  | 1 |  |
| Yes | 0·98  (0·27-3·58) |  | 1·07  (0·61-1·87) |  | 1·06  (0·65-1·74) |  |
| **Non-Chronic disease diagnosed** |  | 0·13 |  | 0·95 |  | 0·50 |
| No | 1 |  | 1 |  | 1 |  |
| Yes | 0·26  (0·05-1·48) |  | 1·03  (0·46-2·30) |  | 0·78  (0·39-1·57) |  |
| **Duration in Thailand** |  | 0·91 |  | 0·31 |  | 0·31 |
| 0-5 years | 1 |  | 1 |  | 1 |  |
| 6-10 years | 0·96  (0·18-5·05) |  | 1·79  (0·84-3·79) |  | 1·64  (0·84-3·18) |  |
| >10 years | 1·23  (0·27-5·55) |  | 1·34  (0·67-2·70) |  | 1·46  (0·79-2·68) |  |
| **Occupation** |  | 0·83 |  | 0·08 |  | 0·24 |
| Physically demanding jobs | 1 |  | 1 |  | 1 |  |
| Less physically demanding jobs | 1·38  (0·42-4·51) |  | 2·45  (1·12-5·36) |  | 1·71  (0·93-3·15) |  |
| Unemployed | 0·98  (0·23-4·17) |  | 1·35  (0·72-2·50) |  | 1·20  (0·70-2·04) |  |
| **Household income** |  | 0·44 |  | 0·06 |  | 0·02 |
| 0-2000 THB | 1 |  | 1 |  | 1 |  |
| 2001-8000 THB | 1·11  (0·18-6·94) |  | 0·44  (0·18-1·06) |  | 0·50  (0·23-1·07) |  |
| 8001-12·000 THB | 2·00  (0·23-17·49) |  | 0·57  (0·22-1·46) |  | 0·67  (0·29-1·52) |  |
| 12·001-30·000 THB | 4·82  (0·44-52·38) |  | 1·50  (0·40-5·61) |  | 1·91  (0·63-5·79) |  |

**Table S8 Model vi) Utilized x-ray/ computed tomography scan or ultrasound: Multivariable analysis of M-FUND membership and socio-demographic variables disaggregated by sex.**

|  | **Men** | | **Women** | | **Total** | |
| --- | --- | --- | --- | --- | --- | --- |
| **M-FUND membership** | **OR**  **(95% CI)** | **p-value** | **OR**  **(95% CI)** | **p-value** | **OR**  **(95% CI)** | **p-value** |
| **Utilized x-ray/ computed tomography scan or ultrasound** |  | 0·18 |  | 0·03 |  | 0·01 |
| No | 1 |  | 1 |  | 1 |  |
| Yes | 3·12  (0·60-16·30) |  | 2·49  (1·08-5·70) |  | 2·62  (1·29- 5·29) |  |
| **District name** |  | 0·71 |  | 0·73 |  | 0·89 |
| Mae Ramat | 1 |  | 1 |  | 1 |  |
| Mae Sot | 2·35  (0·31-18·05) |  | 1·02  (0·44-2·34) |  | 1·12  (0·54- 2·31) |  |
| Phop Phra | 2·03  (0·16-26·10) |  | 1·46  (0·47-4·51) |  | 1·28  (0·48- 3·40) |  |
| **Age** |  | 0·72 |  | 0·99 |  | 0·94 |
| 18-49 | 1 |  | 1 |  | 1 |  |
| >49 | 1·24  (0·39-3·92) |  | 1·00  (0·54-1·86) |  | 1·02  (0·61- 1·69) |  |
| **Sex** |  | ·· |  | ·· |  | 0·32 |
| Male | ·· |  | ·· |  | 1 |  |
| Female | ·· |  | ·· |  | 0·78  (0·48- 1·28) |  |
| **Literacy** |  | 0·22 |  | 0·23 |  | 0·09 |
| No | 1 |  | 1 |  | 1 |  |
| Yes | 3·56  (0·48-26·66) |  | 2·41  (0·57-10·26) |  | 2·62  (0·86- 7·94) |  |
| **Ethnicity** |  | 0·55 |  | 0·88 |  | 0·83 |
| Bamar | 1 |  | 1 |  | 1 |  |
| Karen | 1·30  (0·35-4·75) |  | 0·83  (0·40-1·74) |  | 0·94  (0·51-1·75) |  |
| Other | 2·50  (0·49-12·81) |  | 0·97  (0·44-2·14) |  | 1·16  (0·60- 2·27) |  |
| **Religion** |  | 0·20 |  | 0·83 |  | 0·58 |
| Buddhism | 1 |  | 1 |  | 1 |  |
| Other religion | 0·37  (0·08-1·69) |  | 1·09  (0·50-2·39) |  | 0·83  (0·43- 1·61) |  |
| **Marital status** |  | 0·23 |  | 0·18 |  | 0·07 |
| Single | 1 |  | 1 |  | 1 |  |
| Married | 2·87  (0·79-10·44) |  | 0·90  (0·36-2·26) |  | 1·54  (0·76- 3·12) |  |
| Divorced or widowed | 1·25  (0·12-13·27) |  | 0·48  (0·16-1·45) |  | 0·81  (0·33- 2·04) |  |
| **Legal document** |  | 0·10 |  | 0·46 |  | 0·13 |
| Undocumented | 1 |  | 1 |  | 1 |  |
| Village authority or police paper | 2·07  (0·52-8·32) |  | 1·00  (0·54-1·86) |  | 1·20  (0·70- 2·07) |  |
| Formal legal document | 0·49  (0·16-1·51) |  | 0·70  (0·38-1·29) |  | 0·68  (0·41- 1·13) |  |
| **Educational attainment** |  | 0·14 |  | 0·32 |  | 0·09 |
| None | 1 |  | 1 |  | 1 |  |
| Part of primary | 0·15  (0·02-1·20) |  | 0·25  (0·05-1·16) |  | 0·22  (0·07-0·73) |  |
| Primary/religious | 0·51  (0·06-4·27) |  | 0·24  (0·05-1·26) |  | 0·30  (0·08- 1·04) |  |
| Secondary/high school/vocational school/university | 0·61  (0·07-5·19) |  | 0·21  (0·04-1·11) |  | 0·28  (0·08- 0·98) |  |
| **Chronic disease diagnosed** |  | 0·43 |  | 0·83 |  | 0·61 |
| No | 1 |  | 1 |  | 1 |  |
| Yes | 1·62  (0·49-5·39) |  | 1·06  (0·61-1·85) |  | 1·13  (0·70- 1·84) |  |
| **Non-Chronic disease diagnosed** |  | 0·26 |  | 0·96 |  | 0·50 |
| No | 1 |  | 1 |  | 1 |  |
| Yes | 0·40  (0·08-1·99) |  | 0·98  (0·44-2·20) |  | 0·79  (0·39- 1·58) |  |
| **Duration in Thailand** |  | 0·82 |  | 0·32 |  | 0·30 |
| 0-5 years | 1 |  | 1 |  | 1 |  |
| 6-10 years | 1·27  (0·26-6·29) |  | 1·76  (0·83-3·73) |  | 1·67  (0·86- 3·23) |  |
| >10 years | 1·57  (0·36-6·83) |  | 1·32  (0·66-2·65) |  | 1·46  (0·80- 2·68) |  |
| **Occupation** |  | 0·87 |  | 0·14 |  | 0·39 |
| Physically demanding jobs | 1 |  | 1 |  | 1 |  |
| Less physically demanding jobs | 1·14  (0·35-3·72) |  | 2·22  (1·01-4·84) |  | 1·53  (0·83- 2·80) |  |
| Unemployed | 0·79  (0·19-3·26) |  | 1·31  (0·71-2·42) |  | 1·15  (0·68- 1·95) |  |
| **Household income** |  | 0·58 |  | 0·08 |  | 0·02 |
| 0-2000 THB | 1 |  | 1 |  | 1 |  |
| 2001-8000 THB | 0·85  (0·14-5·16) |  | 0·47  (0·20-1·14) |  | 0·53  (0·25- 1·15) |  |
| 8001-12·000 THB | 0·97  (0·13-7·36) |  | 0·59  (0·23-1·51) |  | 0·68  (0·30- 1·53) |  |
| 12·001-30·000 THB | 3·09  (0·30-31·62) |  | 1·63  (0·44-6·11) |  | 2·02  (0·66-6·13) |  |

**Table S9 Model vii) Utilized blood test: Multivariable analysis of M-FUND membership and socio-demographic variables disaggregated by sex.**

|  | **Men** | | **Women** | | **Total** | |
| --- | --- | --- | --- | --- | --- | --- |
| **M-FUND membership** | **OR**  **(95% CI)** | **p-value** | **OR**  **(95% CI)** | **p-value** | **OR**  **(95% CI)** | **p-value** |
| **Utilized blood test** |  | 0·06 |  | 0·28 |  | 0·05 |
| No | 1 |  | 1 |  | 1 |  |
| Yes | 3·59  (0·98-13·20) |  | 1·39  (0·77-2·50) |  | 1·64  (0·99-2·71) |  |
| **District name** |  | 0·87 |  | 0·75 |  | 0·90 |
| Mae Ramat | 1 |  | 1 |  | 1 |  |
| Mae Sot | 1·67  (0·23-12·40) |  | 1·06  (0·46-2·44) |  | 1·12  (0·54-2·30) |  |
| Phop Phra | 1·81  (0·15-21·25) |  | 1·47  (0·48-4·55) |  | 1·26  (0·48-3·35) |  |
| **Age** |  | 0·66 |  | 0·96 |  | 0·99 |
| 18-49 | 1 |  | 1 |  | 1 |  |
| >49 | 1·30  (0·41-4·15) |  | 0·98  (0·53-1·82) |  | 1·00  (0·60-1·65) |  |
| **Sex** |  | ·· |  | ·· |  | 0·35 |
| Male | ·· |  | ·· |  | 1 |  |
| Female | ·· |  | ·· |  | 0·79  (0·49-1·30) |  |
| **Literacy** |  | 0·26 |  | 0·25 |  | 0·09 |
| No | 1 |  | 1 |  | 1 |  |
| Yes | 3·22  (0·43-24·15) |  | 2·35  (0·55-10·00) |  | 2·61  (0·85-7·99) |  |
| **Ethnicity** |  | 0·37 |  | 0·79 |  | 0·79 |
| Bamar | 1 |  | 1 |  | 1 |  |
| Karen | 1·70  (0·43-6·75) |  | 0·78  (0·37-1·62) |  | 0·91  (0·49-1·68) |  |
| Other Ethnicity | 3·23  (0·63-16·60) |  | 0·95  (0·43-2·08) |  | 1·16  (0·60-2·25) |  |
| **Religion** |  | 0·17 |  | 0·83 |  | 0·54 |
| Buddhism | 1 |  | 1 |  | 1 |  |
| Other religion | 0·35  (0·08-1·55) |  | 1·09  (0·50-2·37) |  | 0·81  (0·42-1·57) |  |
| **Marital status** |  | 0·37 |  | 0·23 |  | 0·09 |
| Single | 1 |  | 1 |  | 1 |  |
| Married | 2·22  (0·61-8·10) |  | 0·89  (0·36-2·21) |  | 1·44  (0·71-2·90) |  |
| Divorced or widowed | 0·90  (0·08-10·25) |  | 0·50  (0·17-1·50) |  | 0·78  (0·31-1·94) |  |
| **Legal document** |  | 0·10 |  | 0·51 |  | 0·12 |
| Undocumented | 1 |  | 1 |  | 1 |  |
| Village authority or police paper | 2·10  (0·52-8·49) |  | 0·98  (0·53-1·82) |  | 1·19  (0·69-2·03) |  |
| Formal legal document | 0·50  (0·16-1·53) |  | 0·71  (0·39-1·31) |  | 0·67  (0·40-1·11) |  |
| **Educational attainment** |  | 0·11 |  | 0·34 |  | 0·09 |
| None | 1 |  | 1 |  | 1 |  |
| Part of primary | 0·14  (0·02-1·19) |  | 0·26  (0·06-1·20) |  | 0·23  (0·07-0·75) |  |
| Primary/religious | 0·59  (0·07-5·26) |  | 0·26  (0·05-1·32) |  | 0·30  (0·08-1·06) |  |
| Secondary/high school/vocational school/university | 0·63  (0·07-5·55) |  | 0·22  (0·04-1·15) |  | 0·28  (0·08-1·01) |  |
| **Chronic disease diagnosed** |  | 0·95 |  | 0·71 |  | 0·65 |
| No | 1 |  | 1 |  | 1 |  |
| Yes | 0·97  (0·26-3·65) |  | 1·11  (0·64-1·93) |  | 1·12  (0·69-1·81) |  |
| **Non-Chronic disease diagnosed** |  | 0·09 |  | 0·98 |  | 0·38 |
| No | 1 |  | 1 |  | 1 |  |
| Yes | 0·22  (0·04-1·29) |  | 0·99  (0·45-2·20) |  | 0·74  (0·37-1·46) |  |
| **Duration in Thailand** |  | 0·66 |  | 0·36 |  | 0·31 |
| 0-5 years | 1 |  | 1 |  | 1 |  |
| 6-10 years | 1·59  (0·30-8·39) |  | 1·71  (0·81-3·61) |  | 1·65  (0·86- 3·18) |  |
| >10 years | 2·02  (0·43-9·41) |  | 1·31  (0·66-2·62) |  | 1·49  (0·81- 2·72) |  |
| **Occupation** |  | 0·94 |  | 0·10 |  | 0·35 |
| Physically demanding jobs | 1 |  | 1 |  | 1 |  |
| Less physically demanding jobs | 1·00  (0·31-3·26) |  | 2·32  (1·07-5·04) |  | 1·55  (0·85-2·83) |  |
| Unemployed | 0·80  (0·20-3·23) |  | 1·34  (0·73-2·47) |  | 1·15  (0·68-1·95) |  |
| **Household income** |  | 0·66 |  | 0·10 |  | 0·03 |
| 0-2000 THB | 1 |  | 1 |  | 1 |  |
| 2001-8000 THB | 1·10  (0·17-7·00) |  | 0·48  (0·20-1·17) |  | 0·55  (0·25-1·17) |  |
| 8001-12·000 THB | 1·21  (0·15-9·68) |  | 0·62  (0·24-1·58) |  | 0·69  (0·30-1·56) |  |
| 12·001-30·000 THB | 3·40  (0·32-36·12) |  | 1·57  (0·42-5·88) |  | 1·87  (0·62-5·66) |  |

**Table S10 Model viii) Utilized urine or other specimen test: Multivariable analysis of M-FUND membership and socio-demographic variables disaggregated by sex.**

|  | **Men** | | **Women** | | **Total** | |
| --- | --- | --- | --- | --- | --- | --- |
| **M-FUND membership** | **OR**  **(95% CI)** | **p-value** | **OR**  **(95% CI)** | **p-value** | **OR**  **(95% CI)** | **p-value** |
| **Utilized urine or other specimen test** |  | 0·22 |  | 0·87 |  | 0·50 |
| No | 1 |  | 1 |  | 1 |  |
| Yes | 0·35  (0·07-1·85) |  | 0·93  (0·41-2·13) |  | 0·78  (0·39-1·59) |  |
| **District name** |  | 0·76 |  | 0·82 |  | 0·95 |
| Mae Ramat | 1 |  | 1 |  | 1 |  |
| Mae Sot | 1·50  (0·22-10·39) |  | 1·07  (0·47-2·45) |  | 1·12  (0·55-2·30) |  |
| Phop Phra | 0·83  (0·07-9·50) |  | 1·39  (0·45-4·28) |  | 1·11  (0·42-2·95) |  |
| **Age** |  | 0·99 |  | 0·94 |  | 0·96 |
| 18-49 | 1 |  | 1 |  | 1 |  |
| >49 | 0·99  (0·32-3·08) |  | 1·02  (0·56-1·88) |  | 1·02  (0·61-1·68) |  |
| **Sex** |  | ·· |  | ·· |  | 0·28 |
| Male | ·· |  | ·· |  | 1 |  |
| Female | ·· |  | ·· |  | 0·76  (0·47-1·25) |  |
| **Literacy** |  | 0·27 |  | 0·25 |  | 0·09 |
| No | 1 |  | 1 |  | 1 |  |
| Yes | 3·08  (0·42-22·57) |  | 2·35  (0·55-10·07) |  | 2·65  (0·87-8·09) |  |
| **Ethnicity** |  | 0·61 |  | 0·77 |  | 0·75 |
| Bamar | 1 |  | 1 |  | 1 |  |
| Karen | 0·87  (0·23-3·35) |  | 0·77  (0·37-1·61) |  | 0·86  (0·47-1·58) |  |
| Other Ethnicity | 1·94  (0·37-10·15) |  | 0·96  (0·44-2·10) |  | 1·11  (0·58-2·16) |  |
| **Religion** |  | 0·23 |  | 0·83 |  | 0·55 |
| Buddhism | 1 |  | 1 |  | 1 |  |
| Other religion | 0·40  (0·09-1·76) |  | 1·09  (0·50-2·37) |  | 0·82  (0·43-1·58) |  |
| **Marital status** |  | 0·18 |  | 0·22 |  | 0·08 |
| Single | 1 |  | 1 |  | 1 |  |
| Married | 3·22  (0·89-11·59) |  | 0·90  (0·36-2·25) |  | 1·55  (0·77-3·12) |  |
| Divorced or widowed | 1·51  (0·13-17·53) |  | 0·50  (0·17-1·50) |  | 0·84  (0·34-2·08) |  |
| **Legal document** |  | 0·10 |  | 0·57 |  | 0·16 |
| Undocumented | 1 |  | 1 |  | 1 |  |
| Village authority or police paper | 2·18  (0·55-8·74) |  | 0·97  (0·52-1·81) |  | 1·16  (0·68- 1·98) |  |
| Formal legal document | 0·52  (0·17-1·56) |  | 0·73  (0·40-1·35) |  | 0·68  (0·41-1·13) |  |
| **Educational attainment** |  | 0·16 |  | 0·33 |  | 0·08 |
| None | 1 |  | 1 |  | 1 |  |
| Part of primary | 0·15  (0·02-1·21) |  | 0·25  (0·05-1·19) |  | 0·22  (0·07-0·73) |  |
| Primary/religious | 0·42  (0·05-3·73) |  | 0·26  (0·05-1·36) |  | 0·30  (0·09-1·07) |  |
| Secondary/high school/vocational school/university | 0·55  (0·06-5·00) |  | 0·22  (0·04-1·15) |  | 0·28  (0·08-1·00) |  |
| **Chronic disease diagnosed** |  | 0·30 |  | 0·61 |  | 0·42 |
| No | 1 |  | 1 |  | 1 |  |
| Yes | 1·96  (0·55-6·90) |  | 1·15  (0·67-2·00) |  | 1·22  (0·75-1·97) |  |
| **Non-Chronic disease diagnosed** |  | 0·16 |  | 0·95 |  | 0·51 |
| No | 1 |  | 1 |  | 1 |  |
| Yes | 0·31  (0·06-1·59) |  | 1·03  (0·46-2·28) |  | 0·79  (0·40-1·57) |  |
| **Duration in Thailand** |  | 0·82 |  | 0·32 |  | 0·29 |
| 0-5 years | 1 |  | 1 |  | 1 |  |
| 6-10 years | 1·19  (0·23-6·02) |  | 1·76  (0·83-3·70) |  | 1·69  (0·88-3·25) |  |
| >10 years | 1·53  (0·35-6·77) |  | 1·30  (0·65-2·59) |  | 1·47  (0·80-2·68) |  |
| **Occupation** |  | 0·87 |  | 0·10 |  | 0·29 |
| Physically demanding jobs | 1 |  | 1 |  | 1 |  |
| Less physically demanding jobs | 1·33  (0·42-4·24) |  | 2·35  (1·08-5·08) |  | 1·61  (0·89-2·93) |  |
| Unemployed | 1·02  (0·25-4·17) |  | 1·37  (0·74-2·52) |  | 1·21  (0·72-2·05) |  |
| **Household income** |  | 0·63 |  | 0·09 |  | 0·03 |
| 0-2000 THB | 1 |  | 1 |  | 1 |  |
| 2001-8000 THB | 0·81  (0·13-4·94) |  | 0·47  (0·20-1·13) |  | 0·52  (0·24-1·10) |  |
| 8001-12·000 THB | 0·67  (0·09-4·96) |  | 0·59  (0·23-1·50) |  | 0·62  (0·28-1·40) |  |
| 12·001-30·000 THB | 2·33  (0·24-22·68) |  | 1·54  (0·41-5·78) |  | 1·76  (0·58-5·31) |  |

**Table S11 Model ix) Utilized specialist consultation: Multivariable analysis of M-FUND membership and socio-demographic variables disaggregated by sex.**

|  | **Men** | | **Women** | | **Total** | |
| --- | --- | --- | --- | --- | --- | --- |
| **M-FUND membership** | **OR**  **(95% CI)** | **p-value** | **OR**  **(95% CI)** | **p-value** | **OR**  **(95% CI)** | **p-value** |
| **Utilized specialist consultation** |  | 0·13 |  | 0·75 |  | 0·28 |
| No | 1 |  | 1 |  | 1 |  |
| Yes | 2·84  (0·73-10·95) |  | 1·11  (0·58-2·12) |  | 1·34  (0·78-2·30) |  |
| **District name** |  | 0·86 |  | 0·80 |  | 0·91 |
| Mae Ramat | 1 |  | 1 |  | 1 |  |
| Mae Sot | 1·66  (0·23-11·79) |  | 1·08  (0·47-2·48) |  | 1·15  (0·56-2·36) |  |
| Phop Phra | 1·32  (0·12-15·11) |  | 1·43  (0·46-4·44) |  | 1·22  (0·46-3·22) |  |
| **Age** |  | 0·84 |  | 0·96 |  | 0·97 |
| 18-49 | 1 |  | 1 |  | 1 |  |
| >49 | 1·12  (0·36-3·50) |  | 1·02  (0·55-1·87) |  | 1·01  (0·61-1·67) |  |
| **Sex** |  | ·· |  | ·· |  | 0·29 |
| Male | ·· |  | ·· |  | 1 |  |
| Female | ·· |  | ·· |  | 0·77 (0·47-1·25) |  |
| **Literacy** |  | 0·23 |  | 0·25 |  | 0·08 |
| No | 1 |  | 1 |  | 1 |  |
| Yes | 3·39  (0·47-24·22) |  | 2·36  (0·55-10·08) |  | 2·69  (0·88-8·20) |  |
| **Ethnicity** |  | 0·51 |  | 0·77 |  | 0·74 |
| Bamar | 1 |  | 1 |  | 1 |  |
| Karen | 1·22  (0·33-4·50) |  | 0·77  (0·37-1·61) |  | 0·87  (0·48-1·60) |  |
| Other Ethnicity | 2·57  (0·51-12·99) |  | 0·96  (0·44-2·11) |  | 1·14  (0·59-2·22) |  |
| **Religion** |  | 0·18 |  | 0·83 |  | 0·54 |
| Buddhism | 1 |  | 1 |  | 1 |  |
| Other religion | 0·36  (0·08-1·59) |  | 1·09  (0·50-2·37) |  | 0·82  (0·42-1·57) |  |
| **Marital status** |  | 0·19 |  | 0·22 |  | 0·08 |
| Single | 1 |  | 1 |  | 1 |  |
| Married | 3·08  (0·86-11·04) |  | 0·90  (0·36-2·25) |  | 1·53  (0·76-3·08) |  |
| Divorced or widowed | 1·26  (0·11-14·78) |  | 0·50  (0·17-1·51) |  | 0·83  (0·33-2·05) |  |
| **Legal document** |  | 0·13 |  | 0·55 |  | 0·14 |
| Undocumented | 1 |  | 1 |  | 1 |  |
| Village authority or police paper | 1·88  (0·46-7·80) |  | 0·99  (0·53-1·83) |  | 1·19  (0·70-2·03) |  |
| Formal legal document | 0·49  (0·16-1·49) |  | 0·73  (0·40-1·33) |  | 0·68  (0·41-1·12) |  |
| **Educational attainment** |  | 0·11 |  | 0·33 |  | 0·09 |
| None | 1 |  | 1 |  | 1 |  |
| Part of primary | 0·12  (0·01-1·00) |  | 0·26  (0·06-1·21) |  | 0·23  (0·07-0·74) |  |
| Primary/religious | 0·46  (0·05-3·83) |  | 0·26  (0·05-1·37) |  | 0·31  (0·09-1·08) |  |
| Secondary/high school/vocational school/university | 0·48  (0·06-4·15) |  | 0·22  (0·04-1·15) |  | 0·28  (0·08-0·98) |  |
| **Chronic disease diagnosed** |  | 0·54 |  | 0·65 |  | 0·54 |
| No | 1 |  | 1 |  | 1 |  |
| Yes | 1·46  (0·44-4·89) |  | 1·13  (0·65-1·97) |  | 1·16  (0·72-1·88) |  |
| **Non-Chronic disease diagnosed** |  | 0·29 |  | 1·00 |  | 0·45 |
| No | 1 |  | 1 |  | 1 |  |
| Yes | 0·42  (0·08-2·12) |  | 1·00  (0·45-2·24) |  | 0·77  (0·38-1·52) |  |
| **Duration in Thailand** |  | 0·77 |  | 0·32 |  | 0·30 |
| 0-5 years | 1 |  | 1 |  | 1 |  |
| 6-10 years | 1·33  (0·27-6·63) |  | 1·75  (0·83-3·69) |  | 1·67  (0·87-3·22) |  |
| >10 years | 1·68  (0·38-7·46) |  | 1·30  (0·65-2·58) |  | 1·46  (0·80-2·67) |  |
| **Occupation** |  | 0·80 |  | 0·09 |  | 0·28 |
| Physically demanding jobs | 1 |  | 1 |  | 1 |  |
| Less physically demanding jobs | 1·23  (0·39-3·89) |  | 2·36  (1·09-5·13) |  | 1·63  (0·90-2·97) |  |
| Unemployed | 0·78  (0·18-3·26) |  | 1·37  (0·74-2·52) |  | 1·19  (0·71-2·02) |  |
| **Household income** |  | 0·70 |  | 0·08 |  | 0·03 |
| 0-2000 THB | 1 |  | 1 |  | 1 |  |
| 2001-8000 THB | 1·02  (0·16-6·29) |  | 0·46  (0·19-1·12) |  | 0·51  (0·24-1·09) |  |
| 8001-12·000 THB | 1·01  (0·13-7·97) |  | 0·58  (0·23-1·49) |  | 0·62  (0·27-1·39) |  |
| 12·001-30·000 THB | 3·03  (0·28-32·62) |  | 1·51  (0·40-5·70) |  | 1·71  (0·57-5·19) |  |

**Table S12 Model x) Utilized treatment for an injury: Multivariable analysis of M-FUND membership and socio-demographic variables disaggregated by sex.**

|  | **Men** | | **Women** | | **Total** | |
| --- | --- | --- | --- | --- | --- | --- |
| **M-FUND membership** | **OR**  **(95% CI)** | **p-value** | **OR**  **(95% CI)** | **p-value** | **OR**  **(95% CI)** | **p-value** |
| **Utilized treatment for injury** |  | 0·12 |  | 0·41 |  | 0·14 |
| No | 1 |  | 1 |  | 1 |  |
| Yes | 3·50  (0·73-16·74) |  | 2·66  (0·26-27·87) |  | 2·36  (0·75-7·44) |  |
| **District name** |  | 0·46 |  | 0·80 |  | 0·95 |
| Mae Ramat | 1 |  | 1 |  | 1 |  |
| Mae Sot | 2·16  (0·28-16·49) |  | 1·08  (0·47-2·48) |  | 1·13  (0·54-2·32) |  |
| Phop Phra | 1·15  (0·10-13·44) |  | 1·43  (0·46-4·41) |  | 1·11  (0·42-2·96) |  |
| **Age** |  | 0·65 |  | 0·97 |  | 0·84 |
| 18-49 | 1 |  | 1 |  | 1 |  |
| >49 | 1·30  (0·41-4·11) |  | 1·01  (0·55-1·86) |  | 1·05  (0·64-1·74) |  |
| **Sex** |  | ·· |  | ·· |  | 0·48 |
| Male | ·· |  | ·· |  | 1 |  |
| Female | ·· |  | ·· |  | 0·83  (0·51-1·38) |  |
| **Literacy** |  | 0·21 |  | 0·25 |  | 0·09 |
| No | 1 |  | 1 |  | 1 |  |
| Yes | 3·72  (0·47-29·22) |  | 2·35  (0·55-10·06) |  | 2·60  (0·85-7·93) |  |
| **Ethnicity** |  | 0·54 |  | 0·78 |  | 0·76 |
| Bamar | 1 |  | 1 |  | 1 |  |
| Karen | 1·16  (0·32-4·22) |  | 0·77  (0·37-1·61) |  | 0·87  (0·47-1·59) |  |
| Other Ethnicity | 2·48  (0·48-12·82) |  | 0·96  (0·44-2·10) |  | 1·12  (0·58-2·17) |  |
| **Religion** |  | 0·16 |  | 0·87 |  | 0·56 |
| Buddhism | 1 |  | 1 |  | 1 |  |
| Other religion | 0·34  (0·08-1·53) |  | 1·07  (0·49-2·33) |  | 0·82  (0·43-1·59) |  |
| **Marital status** |  | 0·16 |  | 0·26 |  | 0·08 |
| Single | 1 |  | 1 |  | 1 |  |
| Married | 3·32  (0·89-12·45) |  | 0·90  (0·36-2·24) |  | 1·57  (0·77-3·18) |  |
| Divorced or widowed | 1·29  (0·12-14·06) |  | 0·52  (0·17-1·55) |  | 0·87  (0·35-2·18) |  |
| **Legal document** |  | 0·08 |  | 0·60 |  | 0·18 |
| Undocumented | 1 |  | 1 |  | 1 |  |
| Village authority or police paper | 2·19  (0·53-9·03) |  | 0·99  (0·53-1·83) |  | 1·18  (0·69-2·03) |  |
| Formal legal document | 0·48  (0·16-1·47) |  | 0·75  (0·41-1·36) |  | 0·70  (0·42-1·16) |  |
| **Educational attainment** |  | 0·10 |  | 0·37 |  | 0·09 |
| None | 1 |  | 1 |  | 1 |  |
| Part of primary | 0·13  (0·02-1·13) |  | 0·26  (0·06-1·24) |  | 0·23  (0·07-0·76) |  |
| Primary/religious | 0·49  (0·06-4·18) |  | 0·27  (0·05-1·38) |  | 0·31  (0·09-1·10) |  |
| Secondary/high school/vocational school/university | 0·69  (0·08-6·23) |  | 0·23  (0·04-1·20) |  | 0·30  (0·08-1·08) |  |
| **Chronic disease diagnosed** |  | 0·42 |  | 0·65 |  | 0·47 |
| No | 1 |  | 1 |  | 1 |  |
| Yes | 1·64  (0·49-5·41) |  | 1·13  (0·66-1·96) |  | 1·20  (0·74-1·93) |  |
| **Non-Chronic disease diagnosed** |  | 0·25 |  | 0·93 |  | 0·56 |
| No | 1 |  | 1 |  | 1 |  |
| Yes | 0·39  (0·08-1·96) |  | 1·04  (0·47-2·29) |  | 0·82  (0·41-1·62) |  |
| **Duration in Thailand** |  | 0·89 |  | 0·30 |  | 0·27 |
| 0-5 years | 1 |  | 1 |  | 1 |  |
| 6-10 years | 1·32  (0·27-6·60) |  | 1·79  (0·85-3·76) |  | 1·71  (0·89-3·30) |  |
| >10 years | 1·45  (0·33-6·37) |  | 1·31  (0·66-2·61) |  | 1·44  (0·79-2·64) |  |
| **Occupation** |  | 0·80 |  | 0·10 |  | 0·29 |
| Physically demanding jobs | 1 |  | 1 |  | 1 |  |
| Less physically demanding jobs | 1·37  (0·43-4·37) |  | 2·34  (1·08-5·08) |  | 1·63  (0·89-2·97) |  |
| Unemployed | 0·92  (0·22-3·78) |  | 1·38  (0·75-2·54) |  | 1·21  (0·72-2·05) |  |
| **Household income** |  | 0·64 |  | 0·08 |  | 0·03 |
| 0-2000 THB | 1 |  | 1 |  | 1 |  |
| 2001-8000 THB | 0·77  (0·12-4·85) |  | 0·47  (0·20-1·13) |  | 0·52  (0·24-1·11) |  |
| 8001-12·000 THB | 0·80  (0·10-6·23) |  | 0·58  (0·23-1·47) |  | 0·63  (0·28-1·41) |  |
| 12·001-30·000 THB | 2·52  (0·24-26·19) |  | 1·55  (0·42-5·76) |  | 1·85  (0·61-5·57) |  |

**Table S13 Model xi) Utilized infusion/injection: Multivariable analysis of M-FUND membership and socio-demographic variables disaggregated by sex.**

|  | **Men** | | **Women** | | **Total** | |
| --- | --- | --- | --- | --- | --- | --- |
| **M-FUND membership** | **OR**  **(95% CI)** | **p-value** | **OR**  **(95% CI)** | **p-value** | **OR**  **(95% CI)** | **p-value** |
| **Utilized infusion/injection** |  | 0·42 |  | 0·44 |  | 0·22 |
| No | 1 |  | 1 |  | 1 |  |
| Yes | 1·56  (0·53-4·60) |  | 1·22  (0·74-2·02) |  | 1·32  (0·85- 2·04) |  |
| **District name** |  | 0·76 |  | 0·81 |  | 0·96 |
| Mae Ramat | 1 |  | 1 |  | 1 |  |
| Mae Sot | 1·63  (0·23-11·47) |  | 1·05  (0·46-2·42) |  | 1·10  (0·53-2·26) |  |
| Phop Phra | 0·95  (0·08-10·72) |  | 1·40  (0·46-4·30) |  | 1·12  (0·43- 2·96) |  |
| **Age** |  | 0·98 |  | 1·00 |  | 0·95 |
| 18-49 | 1 |  | 1 |  | 1 |  |
| >49 | 0·99  (0·32-3·04) |  | 1·00  (0·54-1·85) |  | 0·98  (0·59-1·63) |  |
| **Sex** |  | ·· |  | ·· |  | 0·24 |
| Male | ·· |  | ·· |  | 1 |  |
| Female | ·· |  | ·· |  | 0·75  (0·46-1·22) |  |
| **Literacy** |  | 0·19 |  | 0·27 |  | 0·09 |
| No | 1 |  | 1 |  | 1 |  |
| Yes | 3·89  (0·50-30·04) |  | 2·29  (0·53-9·86) |  | 2·66  (0·87-8·13) |  |
| **Ethnicity** |  | 0·58 |  | 0·73 |  | 0·67 |
| Bamar | 1 |  | 1 |  | 1 |  |
| Karen | 1·09  (0·30-3·90) |  | 0·76  (0·37-1·60) |  | 0·86  (0·47-1·58) |  |
| Other Ethnicity | 2·26  (0·45-11·40) |  | 1·00  (0·45-2·22) |  | 1·17  (0·61- 2·29) |  |
| **Religion** |  | 0·22 |  | 0·85 |  | 0·56 |
| Buddhism | 1 |  | 1 |  | 1 |  |
| Other religion | 0·39  (0·09-1·74) |  | 1·08  (0·50-2·35) |  | 0·82  (0·43-1·59) |  |
| **Marital status** |  | 0·21 |  | 0·21 |  | 0·08 |
| Single | 1 |  | 1 |  | 1 |  |
| Married | 2·80  (0·79-10·00) |  | 0·89  (0·36-2·21) |  | 1·48  (0·74-2·99) |  |
| Divorced or widowed | 1·04  (0·10-11·22) |  | 0·49  (0·16-1·48) |  | 0·80  (0·32-1·98) |  |
| **Legal document** |  | 0·08 |  | 0·54 |  | 0·13 |
| Undocumented | 1 |  | 1 |  | 1 |  |
| Village authority or police paper | 1·99  (0·50-7·95) |  | 0·99  (0·54-1·84) |  | 1·18  (0·69-2·02) |  |
| Formal legal document | 0·46  (0·15-1·41) |  | 0·73  (0·40-1·33) |  | 0·67  (0·41- 1·11) |  |
| **Educational attainment** |  | 0·13 |  | 0·37 |  | 0·08 |
| None | 1 |  | 1 |  | 1 |  |
| Part of primary | 0·13  (0·02-1·06) |  | 0·26  (0·06-1·24) |  | 0·22  (0·07-0·72) |  |
| Primary/religious | 0·43  (0·05-3·61) |  | 0·27  (0·05-1·42) |  | 0·30  (0·09-1·06) |  |
| Secondary/high school/vocational school/university | 0·48  (0·06-4·22) |  | 0·23  (0·04-1·23) |  | 0·28  (0·08-1·01) |  |
| **Chronic disease diagnosed** |  | 0·47 |  | 0·61 |  | 0·48 |
| No | 1 |  | 1 |  | 1 |  |
| Yes | 1·56  (0·47-5·23) |  | 1·15  (0·67-1·99) |  | 1·19  (0·74-1·92) |  |
| **Non-Chronic disease diagnosed** |  | 0·18 |  | 0·89 |  | 0·54 |
| No | 1 |  | 1 |  | 1 |  |
| Yes | 0·32  (0·06-1·66) |  | 1·06  (0·48-2·35) |  | 0·81  (0·41-1·60) |  |
| **Duration in Thailand** |  | 0·85 |  | 0·32 |  | 0·30 |
| 0-5 years | 1 |  | 1 |  | 1 |  |
| 6-10 years | 1·25  (0·25-6·17) |  | 1·76  (0·84-3·70) |  | 1·67  (0·87-3·20) |  |
| >10 years | 1·50  (0·34-6·55) |  | 1·30  (0·65-2·60) |  | 1·46  (0·80-2·66) |  |
| **Occupation** |  | 0·86 |  | 0·09 |  | 0·25 |
| Physically demanding jobs | 1 |  | 1 |  | 1 |  |
| Less physically demanding jobs | 1·34  (0·42-4·27) |  | 2·39  (1·10-5·20) |  | 1·67  (0·91-3·04) |  |
| Unemployed | 1·00  (0·25-3·95) |  | 1·39  (0·75-2·55) |  | 1·23  (0·72-2·08) |  |
| **Household income** |  | 0·64 |  | 0·08 |  | 0·03 |
| 0-2000 THB | 1 |  | 1 |  | 1 |  |
| 2001-8000 THB | 0·89  (0·15-5·41) |  | 0·48  (0·20-1·15) |  | 0·53  (0·25-1·14) |  |
| 8001-12·000 THB | 0·83  (0·11-6·15) |  | 0·61  (0·24-1·56) |  | 0·66  (0·29-1·49) |  |
| 12·001-30·000 THB | 2·78  (0·27-28·18) |  | 1·60  (0·43-5·93) |  | 1·88  (0·62-5·68) |  |
